# Evaluation of Bias-Variance Trade-Off for Commonly Used Post-Summarizing Normalization Procedures in Large-Scale Gene Expression Studies

**DOI:** 10.1371/journal.pone.0099380

**Published:** 2014-06-18

**Authors:** Xing Qiu, Rui Hu, Zhixin Wu

**Affiliations:** 1 Department of Biostatistics and Computational Biology, University of Rochester, Rochester, New York, United States of America; 2 Math Department, DePauw University, Greencastle, Indiana, United States of America; New Jersey Institute of Technology, United States of America

## Abstract

Normalization procedures are widely used in high-throughput genomic data analyses to remove various technological noise and variations. They are known to have profound impact to the subsequent gene differential expression analysis. Although there has been some research in evaluating different normalization procedures, few attempts have been made to systematically evaluate the gene detection performances of normalization procedures from the bias-variance trade-off point of view, especially with strong gene differentiation effects and large sample size. In this paper, we conduct a thorough study to evaluate the effects of normalization procedures combined with several commonly used statistical tests and MTPs under different configurations of effect size and sample size. We conduct theoretical evaluation based on a random effect model, as well as simulation and biological data analyses to verify the results. Based on our findings, we provide some practical guidance for selecting a suitable normalization procedure under different scenarios.

## Introduction

High-throughput data such as microarray expression data and RNA-seq data have become an indispensable tool for medical research nowadays. Typically, a data pre-processing step called *normalization* is conducted prior to the subsequent statistical analyses in order to remove various systematic noise. Pertinent statistical significance tests are applied to these *normalized* gene expression levels. Parametric tests such as Student’s 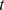-test and its improvements such as the one used in SAM (Significance Analysis of Microarray, [1]) are widely used. Non-parametric tests such as Wilcoxon rank-sum test serve as distribution free alternatives. The resulting *p*-values are adjusted by a multiple testing procedure (MTP) in order to control certain quantity of per-family Type I error, such as familywise error rate (FWER) [2]–[5] and false discovery rate (FDR) [6]. Differentially expressed genes are identified based on a pre-specified threshold of adjusted 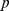-values. More detailed introduction of statistical methods for detecting differentially expressed genes can be found in [7]–[10].

There are different sources of technological noise in high-throughput genomic data [11], [12]. Over the past decade or so, many normalization procedures have been proposed to remove such noise, and they can be loosely categorized into within-array normalizations and multiple-array normalizations. Within-array normalizations remove noise by “borrowing information” from gene expressions within a single array. A simple example is the global normalization, which applies a constant adjustment to force the distribution of gene expressions to have a common mean or median within each array [13], [14]. Another example is the rank normalization which replaces each observation by its fractional rank (the rank divided by the total number of genes) within array [14], [15]. This normalization procedure achieves more robustness to non-additive noise compared with global normalization at the expense of losing some parametric information of expressions.

Multiple-array normalizations adjust the scale across arrays to avoid different arrays having undue weight. A typical example is the quantile normalization. Motivated by quantile-quantile plot, it makes the empirical distribution of gene expressions pooled from each array to be the same [16]. In particular, quantile normalized arrays must have the same sample mean/median. In this sense, it is *stronger* than global normalization. On the other hand, it does not change the rankings of genes and retains more parametric information than the rank normalization. So it can be viewed as a compromise between the global and rank normalization procedures.

A data-driven variable transformation called the 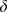-sequence method [17], [18] can serve as an alternative to the aforementioned normalization procedures. In this procedure, essentially each gene is normalized by another one with similar variance which acts as the “reference gene”. This normalization is *local* because only one gene is needed to normalize a given gene. Interested readers are referred to [19], [20] for background and more detailed reviews of normalization procedures.

Surrogate variable analysis (SVA) [21] was designed to overcome the problems caused by heterogeneity in expression data. The rationale of this method is to remove the detrimental effects due to unmodeled variables such as demographic, environmental, and technical factors.

Although SVA was not originally designed as a normalization procedure, it can serve this role as long as the array-specific technical noise is considered as one of the unmodeled factors. To some extend, SVA can be viewed as an extension of the global normalization, which normalizes the the observed expression levels by removing just one factor, per-array mean expressions.

Normalization procedures are designed to remove technological noise and improve the detection of differentially expression genes. However, the testing power improvement from normalization procedures may also come with a price. The performance of a particular normalization procedure in terms of gene selection power and type I error control is heavily influenced by the sample size and true effect size of gene differentiation. Some efforts have been made to evaluate different normalization procedures [16], [22]–[25], but few attempts have been made to systematically evaluate the impact of sample size and effect size on the gene detection performances of normalization procedures.

In this paper, we conduct a thorough study to evaluate the performance of gene selection strategies which consist of four normalization procedures (plus the case without normalization) combined with three statistical tests and two MTPs, with various combinations of sample sizes and effect sizes. It is well-known that variance reduction always comes with the price of bias. We use this bias-variance trade-off principle to study the theoretical statistical properties of normalization procedures. Simulation and biological data analyses are conducted to support the theoretical results in the biological application. Based on our study, we make several concluding points in the Section Discussion. Whenever possible, we provide theoretical explanations to support these conclusions based on a random effect model [15]. We hope these findings can provide biomedical researchers with some practical guidance for selecting the best gene selection strategy.

The following supporting materials are presented in File S1: 1. a brief introduction of the 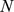-test; 2. theoretical derivations for the main results in the Methods Section; 3. additional simulation results; 4. additional results of biological data analyses.

## Methods

### Model for Gene Expression Data

In this paper, we assume all expression levels are log-tranformed. Additive (non-additive) noise are thus multiplicative (non-multiplicative) noise in terms of the original expression levels. For convenience, the words “gene” and “gene expression” are used interchangeably to refer to these log-transformed random variables.

Let 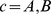 be two different phenotypic groups, 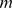 be the total number of genes, and 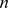 be the number of arrays sampled from each phenotypic group. Without loss of generality, phenotypic group 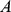 is set to represent the phenotype of interest (usually the disease or the treatment group) and group 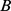 the normal phenotype. So up (down) regulation of a gene refers to its over (under) expression in group 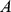. We denote by 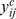 the observed expression level of the 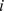
th gene recorded on the 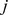
th array sampled from the 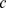
th phenotypic group. Conceptually speaking, the variation of the observed expression, 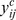, can be decomposed into two sources: a) the biological variation of the 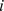
th gene pertain to the 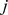
th sample, denoted by 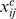 (unobservable); b) the array-specific noise due to imperfect measurement technology, denoted by 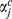 (unobservable).

We denote 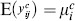 and 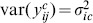. For a given gene, its true effect size, or the expected (geometric) mean difference of expressions between two phenotypes, is written as 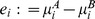. We also assume that the (unobservable) true correlation coefficient between two noise-free expression levels is 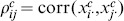 and the correlation coefficient between observed gene expressions 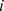 and 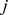 is 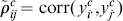. For the biological data (see Section “Biological Data” and [26]) without any normalization procedures, 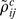 is very high. The sample mean of Pearson correlation coefficients of all gene pairs computed from all three sets of biological data (see Section “Analyzing Biological Data” for more details) are close to 0.9. A histogram of such correlation coefficients is provided as Figure S1 in File S1.

This high correlation may come from two sources [27]: a) the biological correlation of the 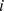
th and 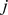
th gene 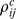; b) the technological noise 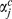.

We divide genes into three sets:

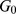
, the set of non-differentially expressed genes (abbreviated as NDEGs). For all 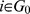, 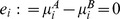;

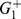
, the set of up-regulated genes. For all 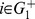, 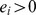;

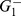
, the set of down-regulated genes. For all 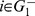, 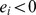.

The set of differentially expressed genes (abbreviated as DEGs) is the union of both up-regulated and down-regulated genes, which is denoted by 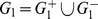. We write the size of these gene sets by 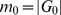, 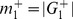, 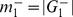, and 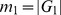. Apparently 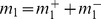 and 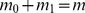.

### Normalization Procedures

The following normalization procedures are studied in this paper:

1. Global normalization (**GLOBAL**): Subtract each element of the data matrix by the mean over all gene expression signals on the array to which this element belongs [13], [14]. The normalized gene expressions are:
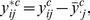
(1)where 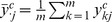 and 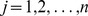.

2. Rank normalization (**RANK**): Replace every gene expression in one array by its rank in the (ascendingly) ordered array divided by the total number of genes. Denote 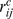 as the rank of 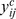 in the array to which it belongs, the normalized gene expressions are.
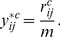
(2)This method was proposed by [15] and discussed further in [14].

3. Quantile normalization (**QUANT**): First, a reference array of empirical quantiles, denoted as 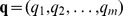, is computed by taking the average across all ordered arrays. Let 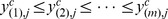 denote the ordered gene expression observations in the 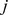
th array (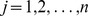) of the 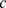
th (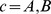) group, the 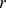
th (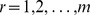) element of this reference array is.
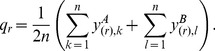
(3)Next, the original expressions are replaced by the entries of the reference array with the same rank. The normalized gene expressions are.
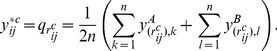
(4)We refer the reader to [16] for more details.

4. 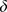-sequence (**DELTA**): Sort all genes by their sample variances and denote the sorted array by 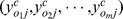, where 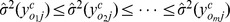. This step is to ensure similar variances between two consecutive genes. Starting with the first gene in the given gene ordering, record the differences between two successive genes:
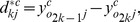
(5)where 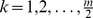. Here 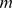 is assumed to be an even number for simplicity. More technical details can be found in [17], [18]. One interpretation of this step is that each gene is normalized by a “reference gene” with similar variance. This normalization is *local* because it only involves one gene to normalize another. Next, select candidate *gene pairs* by applying an appropriate hypothesis test and an MTP to 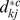. In order to make the 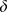-sequence method directly comparable to other methods, the following *ad hoc* method was proposed [17] to “break the pairs”. Starting with the second gene in the given gene ordering, record the differences between two successive genes (the last gene is paired with the first one). Select another set of candidate gene pairs based on these new differences. Report the intersection of the two gene sets (unpaired genes) as a final list of differentially expressed genes. More details can be found in [17], [18].

5. Surrogate variable analyses (**SVA**): in this approach, the observed gene expression is modeled as
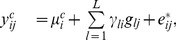
(6)where 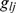 represents an arbitrary function of the 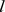
th unmodeled factor on the 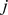
th array, 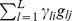 represents dependent variation across genes due to those unmodeled factors, and 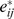 represents the “true” independent noise that are specific to the 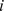
th gene and 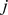
th array. The designing goal of SVA is to estimate and remove 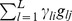 from the observed expression levels, so that the subsequent statistical analysis will not be influenced by those unmodeled factors.

Since 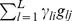 is typically not directly observable, SVA uses the following alternative model to remove the effects of unmodeled factors
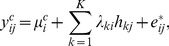
(7)where 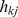, 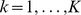, 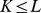, are orthogonal vectors that span the same linear subspace as 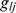
s do.

Gene differential analysis based on SVA is typically done in this way. In the first step, expression levels are fitted by two linear models. The first one is a null model which only includes the surrogate variables; the second one is a full model which includes both surrogate variables and group labels. Two choices are available: a) ordinary linear regression based on least squares; b) a modified linear regression method implemented in LIMMA. An 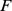-test can then be used to test whether the group labels are significant. More technical details can be found in [21].

Due to the nature of the SVA method, a combination of SVA with linear regression is comparable to the two-sample 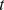-test together with other normalization methods; SVA/LIMMA is comparable to LIMMA with other normalization procedures. Since the SVA method is strongly tied with linear regression methods, we chose not to combine Wilcoxon rank-sum test and 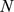-test with it in this study.

6. As a comparison, we also study the properties of gene selection procedures without normalization (**NONE**).

### The Bias-variance Trade-off of Normalization Methods

The choice of normalization procedure has a profound impact on the subsequent analyses. It can alter both the testing power and type I error of the gene selection procedure. In this section, we evaluate the impact of various normalization procedures on testing power and type I error control from the view point of bias-variance trade-off.

To simplify theoretical derivation, we assume that the mean expression levels in the normal phenotype (group 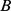) are zeros (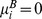). This assumption implies that 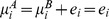. This simplification is reasonable because all three hypothesis testing procedures studied in this paper (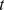-test, Wilcoxon rank-sum test, and 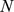-test) are invariant under shift transformation, so the mean difference between two groups, 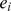, is much more important than the normal level of gene expressions. For derivation simplicity, we also assume that the effect sizes of all up(down)-regulated genes are the same. In summary, we have
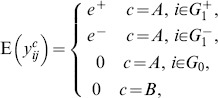
(8)where 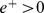 and 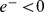 are the effect sizes of up- and down-regulated genes.

Below we list the bias increase and variance reduction induced by normalization procedures. Theoretical derivations of these results can be found in Section 2 of File S1.

#### Global normalization

By computing the expectation of global normalized expressions for both phenotypes, we find that the global normalization introduces a small bias 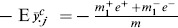. More specifically, the expected mean group difference for global normalized data are
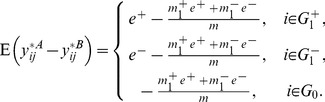
(9)This is because genes are normalized with respect to the array means, which is computed from both DEGs and NDEGs.

**GLOBAL** can reduce the variance of gene expression. Specially, if 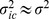 and 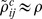 for all genes, we have
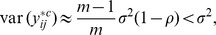
which shows that the global normalization can reduce the variance of expressions, especially when 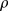 is close to 1.

This variance reduction is confirmed in both simulations and biological data analyses. In **SIMU3** (see Section Simulation Studies for more details) with sample size 10 and effect size 1.8, the average sample variance of expression levels is 0.1286 before global normalization and 0.01367 after. For the biological data **HYPERDIP** with sample size 10, the average sample variances before and after global normalization are 0.1514 and 0.01395, respectively.

When 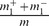, the proportion of DEGs among all genes, is sufficiently small, the bias introduced by the global normalization is negligible because
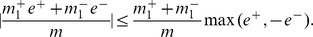
Alternatively, this bias can be negligible if a) the numbers of up and down regulated genes are similar (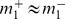); b) up and down regulations induce similar differential expression (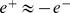). We call this case the *balanced differential expression structure* henceforth. In either case, we predict that the testing power will be improved because the induced bias is small and the variances of global normalized genes are reduced.

The bias effect can be substantial if the differential expression structure is highly unbalanced. For example if most DEGs are up-regulated (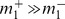, 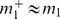), the mean expression difference for an up-regulated gene is reduced by 
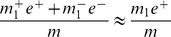
 after the global normalization. Based on these considerations, we predict that the testing power in the balanced structure case will be better than that in the unbalanced structure case.

From Equation 9, a false effect size 
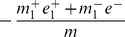
 is introduced by **GLOBAL** for the NDEGs. This false effect size can become substantial when the effect size or the sample size increases. As a result, we predict that many NDEGs will be falsely declared as DEGs. We also predict that the resulted false positives are more pronounced in unbalanced structure than in balanced structure.

#### Quantile normalization

Like the global normalization, the quantile normalization can also increase bias and reduce variance of the gene expressions simultaneously.

We conduct thorough investigation of this bias and discover that it comes from two different sources, namely the rank skewing effect and the averaging effect (see Section 2, File S1). Based on our derivations, the expected group differences for quantile normalized expressions are
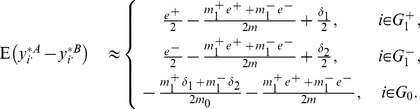
(10)where 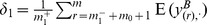 and 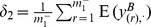.

The effect size of DEGs decreases if 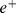 and 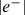 are substantially larger than both 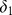 and 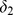. Such effect size decrease is confirmed in our simulation. Take **SIMU3** (see Section Simulation Studies for more details) with sample size 10 and true effect size 1.8 as an example, the average sample mean difference 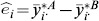 for up(down)-regulated DEGs is 1.803 (–1.7966) before quantile normalization and 0.9901 (–1.0383) after. The average sample mean differences for NDEGs before and after quantile normalization are 0.0034 and −0.0198, respectively.

The variance of 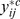 is complex and deserves further theoretical investigation. Empirical evidence shows that the quantile normalization has very good variance reduction capability. For example, the average sample variances of **SIMU3** with sample size 10 and effect size 1.8 before and after quantile normalization are 0.1286 and 0.06378, respectively. Similarly, the average sample variances of biological data (**HYPERDIP**, sample size 10) before and after quantile normalization are 0.1514 and 0.0086, respectively. The variance reduction of **GLOBAL** is better than that of **QUANT** in **SIMU3** since **GLOBAL** is designed for additive noise structure. On the other hand, the variance reduction of **QUANT** is better than that of **GLOBAL** in real biological data since **QUANT** is robust to non-additive noise structure.

**Figure 1:**
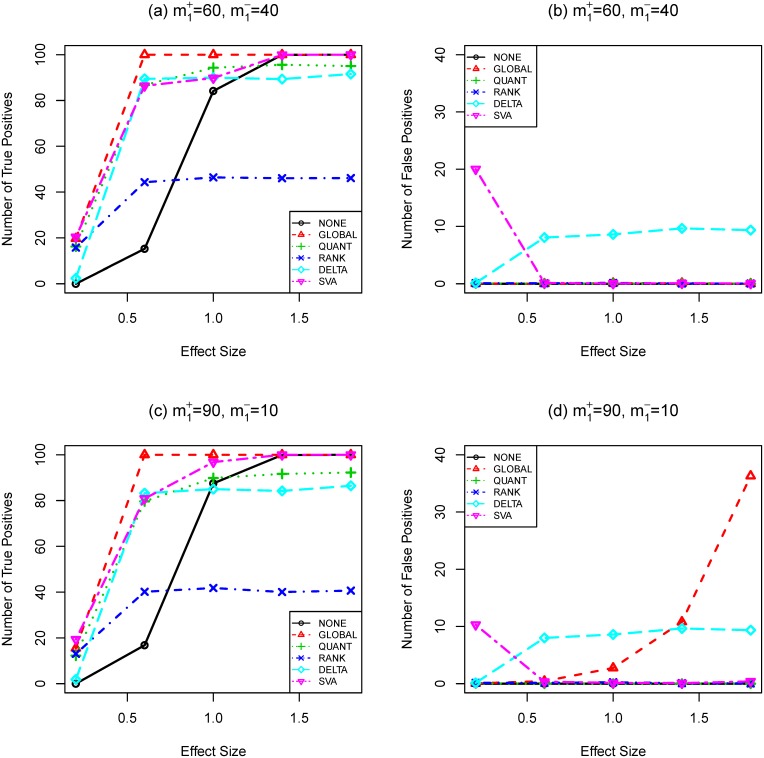
Number of true (a,c) and false (b,d) positives as functions of effect size (SIMU1). Total number of genes is 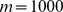. Total number of truly differentially expressed genes is 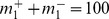, where 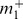 and 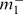 are the numbers of up- and down-regulated genes, respectively. The sample size is 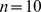. *t*-test and Bonferroni procedure are applied. Adjusted 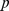-value threshold: 0.05.

**Figure 2:**
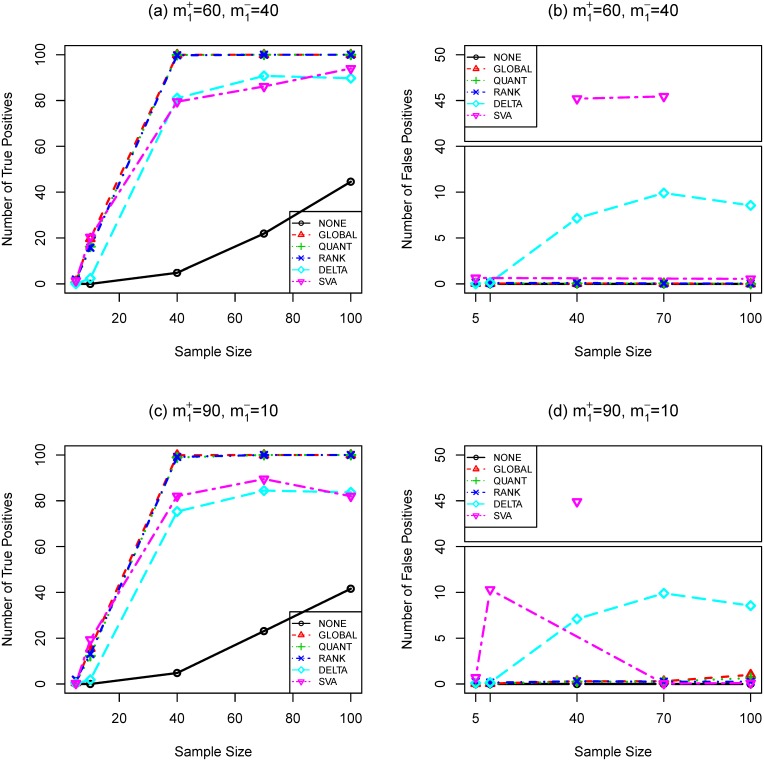
Number of true (a,c) and false (b,d) positives as functions of sample size (SIMU2). Total number of genes is 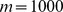. Total number of truly differentially expressed genes is 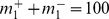, where 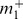 and 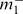 are the numbers of up- and down-regulated genes, respectively. The effect size is 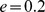. *t*-test and Bonferroni procedure are applied. Adjusted 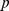-value threshold: 0.05.

**Figure 3:**
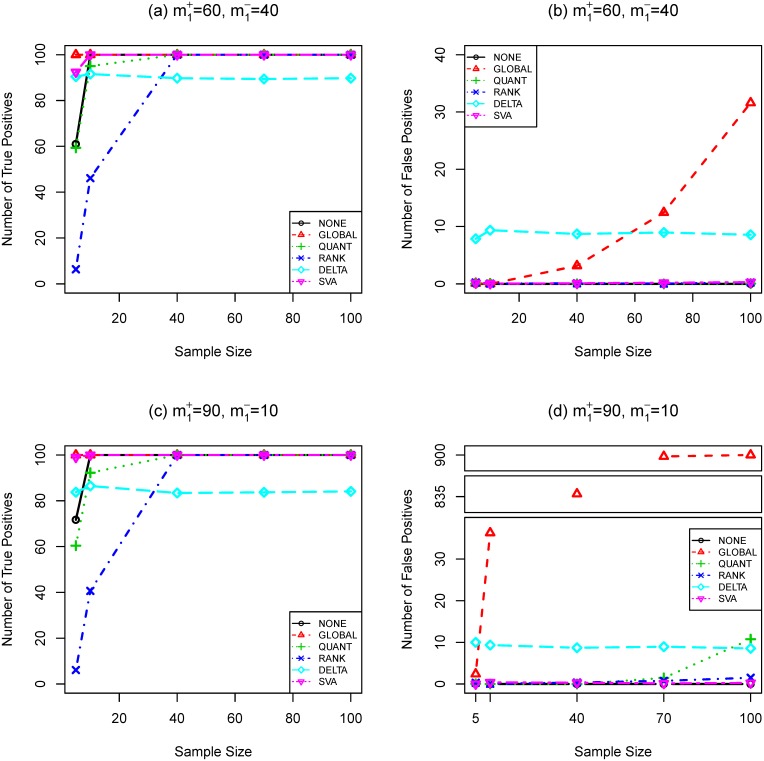
Number of true (a,c) and false (b,d) positives as functions of sample size (SIMU3). Total number of genes is 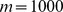. Total number of truly differentially expressed genes is 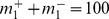, where 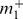 and 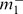 are the numbers of up- and down-regulated genes, respectively. The effect size is 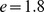. *t*-test and Bonferroni procedure are applied. Adjusted 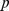-value threshold: 0.05.

Like the global normalization, the benefit of variance reduction induced by the quantile normalization outweighs the adverse effect of bias introduced by this procedure when 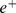, 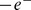, and 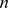 are small. This can be seen from simulation results in Figures 1, 2, and 3(a, c). Take Figure 1 (a, c) as an example, the testing power with quantile normalization increases as the effect size increases given that the sample size is small. However, gene selection strategies based on the quantile normalization is outperformed by those without normalization when effect size reaches a certain point (around effect size 1.4).

According to Equation (6) in File S1 and Equation (10), the effect size of DEGs is reduced for both balanced and unbalanced differential structures. For DEGs, their effect size after quantile normalization has three parts: a) 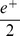 (up-regulated) or 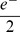 (down-regulated); b) 
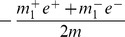
; c) 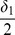 (up-regulated) or 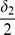 (down-regulated). The first part is clearly independent of the structure of differential expression. The third part is small as compared to the other two parts according to Equation (6) in File S1. The second part is negligible for balanced differential structure but can be substantial for unbalanced differential structure. For example, if 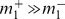, 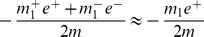. Consequently, we predict that the testing power for data with balanced differential structure is better than those with unbalanced differential structure.

For NDEGs, the quantile normalization introduces a bias 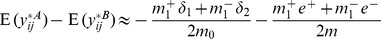 based on Equation (10). This bias is more pronounced in an unbalanced structure. With large effect size or sample size, this bias can lead to nontrivial false positives.

#### Rank normalization

Compared with the quantile normalization, the rank normalization goes even further in the nonparametric direction. Based on derivations in Section 2 of File S1, the expected group expression differences for rank normalized expressions have the following approximation
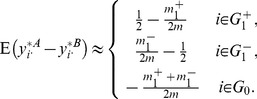
(11)From Equation (11), **RANK** introduces a bias 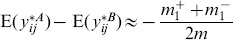. This small bias is nonzero in an unbalanced structure, which can lead to nontrivial false positives when the sample size is large.

In a highly unbalanced gene differential expression structure, for example 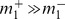, the expected difference for up-regulated genes is approximately 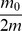 which is smaller than the expected difference in the balanced structure (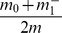). Therefore we predict that the testing power in a balanced structure is higher than that in an unbalanced structure.

The variance reduction effect of **RANK** comes from a very different mechanism as compared to the other normalization procedures. It removes the variance of noise by only preserving the ordering of observations.

#### 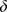-sequence method

The bias increasing effects of **DELTA** comes from the imperfect “pair-breaking” method. Due to the nature of **DELTA**, a pairing breaking method must be used to produce list of individual DEGs. Specifically, a gene is labeled as DEG only if it belongs to two significant gene pairs. On average, the probability of a given NDEG to be paired with a DEG twice is approximately 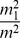. When this relatively rare event happens, an artificial bias will very likely cause it to be mis-classified as DEG [17], [18]. This effect is confirmed in the simulation studies.

The variance reduction effects of **DELTA** come from the gene pairing and subtraction. This variance reduction is confirmed in both simulation studies and real data analyses. As an example, the average sample variances of data **SIMU3** with sample size 10 and true effect size 1.8 before and after 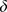-sequence procedure are 0.1224 and 0.0232, respectively. Similarly, the average sample variances of biological data (**HYPERDIP**, sample size 10) before and after 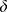-sequence procedure are 0.1514 and 0.0237, respectively. Figures 1 and 2 (a, c) show that the 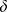-sequence method improves the testing power compared with the non-normalized case when the sample size and effect size are both small, but the improvement is not as good as other normalization procedures. In a balanced structure, the expected expression difference can be as large as 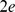 compared to the maximum expected difference 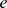 in an unbalanced structure. Thus the testing power in a balanced structure is better than that in an unbalanced structure, *ceteris paribus*. When the sample size or effect size becomes large, the testing power of 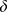-sequence method can only reach approximately 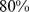, which is determined by 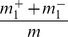, the proportion of DEGs among all genes. More details can be found in [17], [18].

Unlike other normalization procedures (**GLOBAL**, **QUANT**, and **RANK**), we predict that strategies based on **DELTA** produce a stable number of false positives approximately equal to 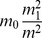, which is *independent of* either the sample size or the effect size. This property of **DELTA** can be attractive when the effect size or the sample size is large.

#### Surrogate variable analysis method

Since the SVA method is based on a complex linear model (Equation (7)), direct derivation of mathematical expectation and variance of normalized expressions are difficult and deserve further investigation. Empirical evidences show that when combined with differential expression analysis, SVA increases statistical power while introduce more false positives. This empirical finding is consistent with the bias-variance trade-off effect of other normalization procedures.

### Hypothesis Testing Methods and Multiple Testing Procedures

We apply the following tests to the normalized data to compute unadjusted 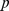-values (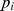, 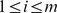):

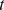
-test (**t**).
2. A moderated 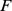-test implemented in R/BioConductor package LIMMA [28] (**LIMMA**).
3. Wilcoxon rank-sum test (**Wilcox**).

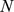
-test, a permutation test based on 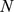-statistics with Euclidean kernel (**Nstat**).

The third test is a multivariate nonparametric test which has been successfully used to select differentially expressed genes and gene combinations [14], [29]–[31], differentially associated genes [32], [33], and synergistic modulators [34]. A brief introduction of the 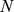-test can be found in Section 1 of File S1.

In order to control Type I errors, a suitable multiple testing procedure (MTP) must be applied to 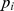 to compute adjusted 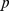-values. The following two widely used MTPs are employed in this paper:

1. Bonferroni procedure (**BONF**): The adjusted 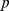-values are 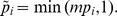 This procedure controls the familywise error rate (FWER).
2. Benjamini-Hochberg procedure (**BH**): Let 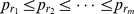 be the ordered raw 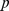-values. The adjusted 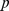-values are 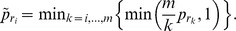 This procedure controls FDR, the false discovery rate [6].

We present our results based on 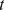-test and Bonferroni procedure in the main text. The results of other hypothesis testings and MTPs are similar and provided in Tables S1–S9 of File S1.

### Biological Data

The biological dataset used in this study is the childhood leukemia dataset from the St. Jude Children’s Research Hospital database [26]. We select three groups of data: 88 patients (arrays) with hyperdiploid acute lymphoblastic leukemia (**HYPERDIP**), 79 patients (arrays) with a special translocation type of acute lymphoblastic leukemia(**TEL**) and 45 patients (arrays) with a T lineage leukemia (**TALL**). Since the original probe set definitions in Affymetrix GeneChip data are known to be inaccurate [35], we update them by using a custom CDF file to produce values of gene expressions. The CDF file was downloaded from http://brainarray.mbni.med.umich.edu. Each array is represented by an array reporting the logarithm (base 2) of expression level on the set of 9005 genes.

## Results

### Simulation Studies

To match the statistical properties of real gene expression more closely and mimic other noise sources such as non-additive noise, we apply resampling method to the biological data to construct the main simulated data, denoted by **SIMU-BIO**, as follows. We apply 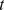-test to **HYPERDIP** and **TEL** (79 slides chosen from each set) without any normalization procedure or multiple testing adjustment. Under the significance level 0.05, 734 genes are declared to be DEGs with an unbalanced differential expression structure (677 up-regulated and 57 down-regulated). We record the mean difference across **HYPERDIP** and **TEL** for each DEG as its effectwww.ixinyiwu.com size (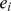). Then, we combine **HYPERDIP** and **TEL** data and randomly permute the slides. After that, we randomly choose 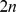 slides and divide them into two groups 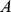 and 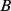 of 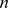 slides each, mimicking two biological conditions without differentially expressed genes. Here the sample size 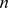 takes value in 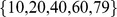. Finally, we add the recorded effect sizes to 734 genes (identified earlier) in group A. These 734 genes are defined as the DEGs in this simulation. Similarly, we test phenotypic differences between **TALL** and **TEL** (45 slides chosen from each set) and discover 546 DEGs with a balanced differential expression structure (259 up-regulated and 287 down-regulated). We then apply the above resampling procedure to create simulated data with sample size 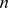 takes value in 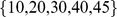.

In addition, we conduct several simulation studies based on a widely adopted random effect model used in [15], [36]–[38]:
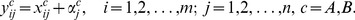
(12)In this model, 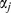 represents variation that is the same for every gene and specific to the 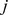
th array. While it is known that log transformation stabilizes variance for microarray data, more advanced variance stabilization transformation techniques [39], [40] can achieve better uniformity of gene-specific variation.

These simulated data can help us to gain better insight into the performance of different normalization procedures. First, we simulate several sets of data with additive noise based on a Each set of data has two groups of 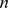 arrays representing gene expressions under two phenotypic groups (group 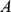 and 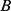). Each array has 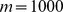 genes. For both groups, all genes are normally distributed with standard deviation 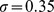 which is estimated from the biological data. The number of NDEGs and DEGs are set to be 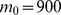 and 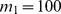, respectively. The correlation coefficient between every two distinct genes is set to be 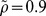, which is estimated from the biological data. For simplicity, we use the same effect size for up and down regulation (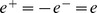).

We generate three sets of simulated data with the following configurations.

- **SIMU1:** The expectations of DEGs in group 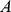 (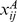, 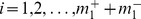, 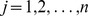) are set to be a constant 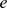 for over-expressed genes (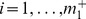) and 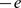 for under-expressed genes (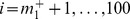). Here the effect size 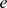 takes value in 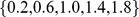. 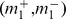 is set to be either 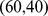 (balanced differential expression structure) or 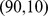 (unbalanced differential expression structure). The lower and upper bounds 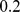 and 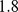 are both estimated from the biological data, so are the proportions of over and under-expressed genes. For all genes in group 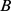 and NDEGs in group 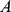, their expectations are set to be 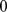. We use **SIMU1** to study the impact of different effect sizes on gene normalization procedures when the sample size is fixed and relatively small. 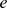 is the tuning parameter of these data sets.
- **SIMU2:** The expectations of DEGs in group 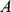 are set to be 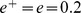 and 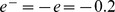 for over and under-expressed genes, respectively. 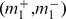 is set to be 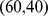 and 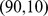. For all genes in group 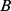 and NDEGs in group 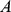, their expectations are set to be 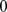. The sample size 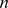 takes value in 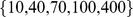. We use **SIMU2** to study the impact of different sample sizes on gene normalization procedures when the effect size is fixed and relatively small. 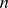 is the tuning parameter of these data sets.
- **SIMU3:** These datasets have the same configuration as **SIMU2** except that the expectations of DEGs in group 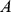 are set to be 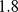 and 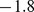 for over and under-expressed genes, respectively. We use **SIMU3** to study the impact of different sample sizes on gene normalization procedures when the effect size is fixed and relatively large. 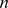 is the tuning parameter of these data sets.

**Figure 4:**
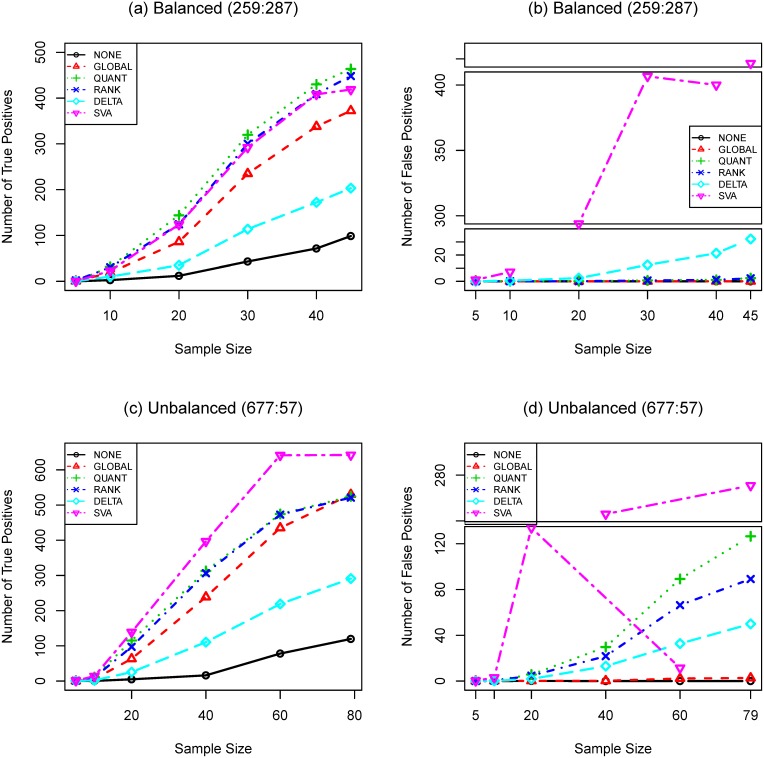
Number of true (a,c) and false (b,d) positives as functions of sample size (SIMU-BIO). Total number of genes is 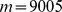. Total numbers of truly differentially expressed genes are 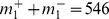 for balanced structure and 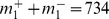 for unbalanced structure, where 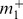 and 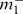 are the numbers of up- and down-regulated genes, respectively. *t*-test and Bonferroni procedure are applied. Adjusted 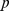-value threshold: 0.05.

**Figure 5:**
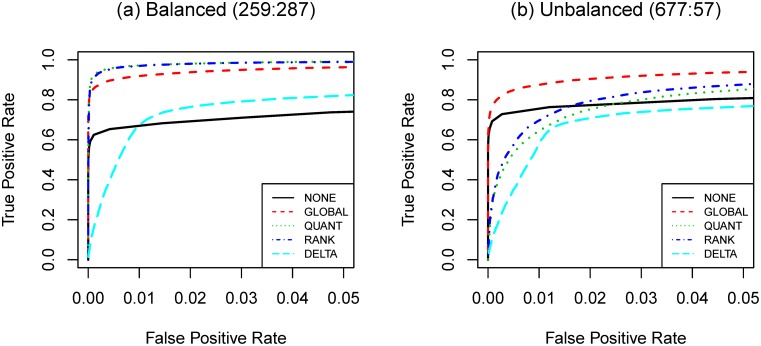
Comparing the performance of normalization procedures by receiver operating characteristic curves. Data used: **SIM-BIO** with 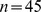 for (a) and 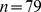 for (b). Total number of genes is 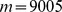. Total numbers of truly differentially expressed genes are 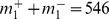 for balanced structure and 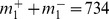 for unbalanced structure, where 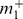 and 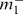 are the numbers of up- and down-regulated genes, respectively. *t*-test and Bonferroni procedure are applied.

We randomly generate 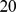 sets of data per tuning parameter for **SIMU-BIO**, **SIMU1**, **SIMU2**, and **SIMU3**. We apply normalization procedures first and then conduct hypothesis tests to obtain raw *p*-values. After that, we apply multiple testing procedures to get adjusted *p*-values. We declare a gene to be differentially expressed if its adjusted *p*-value is less than a prespecified significance level 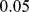. The estimated average true/false positives of each normalization procedure with 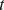-test and **BONF** MTP are presented in Figures 4, 1, 2, and 3. To better understand the trade-off between true and false positives, receiver operating characteristic (ROC) curves of gene selection strategies based on different normalization methods are presented in Figure 5. More thorough results are presented as Tables S1–S8 in File S1.

### Analyzing Biological Data

**Figure 6:**
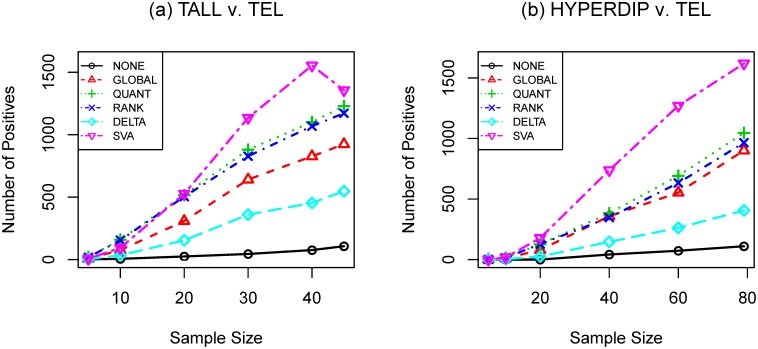
Number of detected DEGs as a function of sample size. (a): **TALL** versus **TEL**; (b) **HYPERDIP** versus **TEL**. Total number of genes is 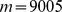. *t*-test and Bonferroni procedure are applied. Adjusted 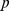-value threshold: 0.05.

We apply the aforementioned gene selection strategies to detect differentially expressed genes across three different microarray datasets (**HYPERDIP**, **TEL** and **TALL**). The numbers of positives with 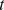-test and **BONF** are presented in Figure 6 and Table S9 in File S1.

Most results agree with what we observe in the simulation studies. The results are conspicuous in that the numbers of detected DEGs become very large when 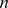 is large (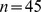 or 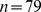). Such a large number of positives may indicate the associated strategies failed to control the familywise error rate at its nominal level (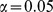).

As observed in the simulation studies, gene selection strategies with normalization procedures detect more DEGs than those without normalization. For **TALL** vs. **TEL**, the strategies with **QUANT** and **RANK** detect more DEGs than those with **GLOBAL**. This observation suggests that the technical noise may not be purely additive and is consistent with what we observe in **SIMU-BIO**. Among four normalization procedures, **SVA** produces the largest number of postives and **DELTA** is the most conservative one. Based on our simulation results, we think it is reasonable to believe that the 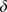-sequence method has relatively better control of type I errors.

## Discussion

It is well known that many undesirable systematic variations are observed in high-throughput genomic data. There are many choices of normalization procedures to remove systematic noise. The properties of these normalization procedures are closely related to the structure of differential gene expression and sample size.

In this study, we find that all four normalization procedures can reduce the variances and covariances of gene expressions so that the statistical power of the subsequent gene selection procedures may be improved. However, they also introduce certain biases which may cause more Type I errors and/or reduce testing power in certain situations. This bias-variance trade-off is common in many different branches of statistics. We found that gene selection strategies based on **GLOBAL** seem to have the best testing power for data generated from a Gaussian model. However, when the effect size is large, they produce far more false positives than which are permitted by the nominal significance level of Type I errors, especially when the gene differential expression structure is unbalanced (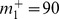, 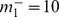). Gene selection strategies based on **QUANT** and **RANK** still have good control of Type I error while retaining reasonable testing power. In addition, sometimes they help detect more DEGs in **SIMU-BIO**, which suggests that these two normalizations work better than **GLOBAL** when the technical noise is not entirely additive (in the log-scale). Overall, gene selection strategies based on **NONE** (without normalization) have very good control of type I errors, but their statistical power are poor and the variability of results (in terms of the standard deviations of the true/false discoveries) are larger. One explanation of this phenomenon is that all normalization procedures reduce the variability caused by the 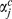 term, which quantifies the array-specific variation, from Model (12), thus increase the signal-to-noise ratio of the normalized expressions. It is known [27] that large 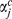 term induces high intergene correlation. Consequently, when the intergene correlation of non-normalized expressions is low, **NONE** has better statistical power. As a comparison, we reduced 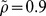 to 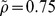 in **SIMU1** and applied the same gene selection strategies as before. The true/false positives produced by gene selection strategies based on normalized data are almost identical to those produced from the original **SIMU1** data; but **NONE** has slightly better power. For example, with true effect size 1.0 and 60 up regulated genes, the mean number of true positives detected by 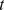-test and Bonferroni procedure increases from 84.15 to 86.95.

Among all normalization procedures, we found that **DELTA** is the most robust one. The numbers of false positives produced by the 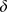-sequence are very consistent for all sample sizes and effect sizes. Its testing power never reaches 100%, however. As a comparison, gene selection strategies without normalization have better performance in terms of testing power and Type I error control compared with their counterparts with normalization when the sample size is large and/or the effect size is large.

Compared with other normalization procedures, **SVA** produces very unstable results. Take Figure 4 (**SIMU-BIO**) as an example, the testing power related with **SVA** is very good (especially for the unbalanced data), but it produces far too many false positives in several occasions. Similar unstable behavior of **SVA** can be observed in Figures 1, 2, and 3.

Another notable result is that gene selection procedures based on normalizations have better power while retaining less or comparable false positives when the gene differential expression structure is balanced.

We also compare the performance of different normalization procedures by ROC curves. ROC curves represent the balance of true and false positives detected at different significance level and is most relevant if the primary goal of expression analysis is to select a fixed number of “top genes” instead of using a formal MTP to control type I error. In Figure 5, **QUANT** and **RANK** have the best performance when the differential expression structure is balanced; **GLOBAL** is the clear winner otherwise. **NONE** is more attractive when the differential expression structure is unbalanced. **DELTA** has poor performance compared with other options. **GLOBAL** seems to have the best overall performance for both cases.

Based on these results, our main conclusions can be summarized as follows.

1. We recommend applying normalization when the sample size is relatively small (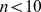 per-group). Failing to do so may lead to dismal statistical power and high variability of results. Cautions still need to be used in this case because some normalization procedures are more susceptible to large phenotypic changes and/or non-additive noise. If unsure, we recommend quantile or rank normalization because they are more robust to these factors.
2. We only recommend global normalization if either a) the statistical power is too low (too few DEGs are identified) and the differential expression structure (in terms of up/down regulated genes) is balanced; b) the goal is to select a fixed number of “top genes”.
3. For large sample data, we recommend conducting differential expression analysis without normalization first. If the statistical power is adequate and a reasonably high percentage (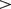
5%) of genes are selected as DEGs, we recommend no further normalization. This is because if the power is adequate, the advantage of variance-reduction provided by normalization procedures could be out-weighted by the bias and thus the inflation of false positives induced by these procedures. If the statistical power is too low, a robust normalization such as rank or 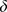-sequence normalization is recommended.
4. The 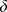-sequence method can serve as a robust normalization candidate when either sample size is large or dramatic phenotypic changes are expected.

We think similar analysis can be applied to models which characterize systematic noise sources in other ways. Though we choose to focus on the Affymetrix GeneChip platform throughout this paper, our conclusions should be valid for other array platforms which require/recommend normalization, such as Affymetrix exon-arrays [41], [42], Illumina BeadChip arrays [43]–[45], Illumina transcriptome sequencing (mRNA-Seq) data [46], Illumina Infinium whole genome genotyping (WGG) arrays [47], Solexa/Illumina deep sequencing technology [48], and many others.

In a sense, between-array variation can be considered as a special form of batch-effect, in which one “batch” consists of just a single array. In this way, several pre-processing procedures that are designed to remove batch effects, such as RUV-2 [49], ComBat [50], can be used in place of normalization procedures. Further investigations are required to fully understand the utility of these procedures when used for normalization, especially in large-scale studies.

We focus on post-summarizing normalization procedures in this study because it is easier to derive the asymptotic bias and variance formula based on a random effect model. In practise, many normalization procedures are applied before the summarization step, which makes theoretical derivation of the bias-variance trade-off difficult. Nevertheless, we think the same principle can be applied to those normalization procedures and further investigations are warranted. We hope this study can help biological researchers choose an appropriate gene selection procedure. Understanding both advantages and disadvantages of different gene selection strategies may also help the development of new normalization procedures, hypothesis tests and MTPs.

## Supporting Information

File S1
**Supporting tables and figures. Table S1.** The impact of different effect sizes 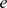 on gene selection strategies when the sample size 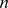 is fixed and relatively small. Mean (STD) of **true positives** computed from **SIMU1** with 20 repetitions are reported. Sample size: 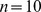. Total number of genes: 1000. Number of differentially expressed genes: 100. Number of permutations for Nstat: 10000. The significance threshold: 0.05. **Table S2.** The impact of different effect sizes 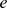 on gene selection strategies when the sample size 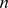 is fixed and relatively small. Mean (STD) of **false positives** computed from **SIMU1** with 20 repetitions are reported. Sample size: 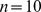. Total number of genes: 1000. Number of differentially expressed genes: 100. Number of permutations for Nstat: 10000. The significance threshold: 0.05. **Table S3.** The impact of different sample sizes 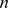 on gene selection strategies when the effect size 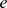 is fixed and relatively small. Mean (STD) of **true positives** computed from **SIMU2** with 20 repetitions are reported. Effect size: 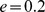. Total number of genes: 1000. Number of differentially expressed genes: 100. Number of permutations for Nstat: 10000. The significance threshold: 0.05. **Table S4.** The impact of different sample sizes 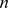 on gene selection strategies when the effect size 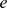 is fixed and relatively small. Mean (STD) of **false positives** computed from **SIMU2** with 20 repetitions are reported. Effect size: 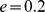. Total number of genes: 1000. Number of differentially expressed genes: 100. Number of permutations for Nstat: 10000. The significance threshold: 0.05. **Table S5.** The impact of different sample sizes 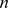 on gene selection strategies when the effect size 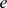 is fixed and relatively large. Mean (STD) of **true positives** computed from **SIMU2** with 20 repetitions are reported. Effect size: 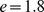. Total number of genes: 1000. Number of differentially expressed genes: 100. Number of permutations for Nstat: 10000. The significance threshold: 0.05. **Table S6.** The impact of different sample sizes 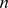 on gene selection strategies when the effect size 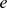 is fixed and relatively large. Mean (STD) of **false positives** computed from **SIMU2** with 20 repetitions are reported. Effect size: 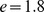. Total number of genes: 1000. Number of differentially expressed genes: 100. Number of permutations for Nstat: 10000. The significance threshold: 0.05. **Table S7**. The impact of different sample sizes 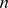 on gene selection strategies with simulation based on biological data. Mean (STD) of **true positives** computed from **SIMU-BIO** with 20 repetitions are reported. Total number of genes: 9005. Number of permutations for Nstat: 100000. The significance threshold: 0.05. **Table S8.** The impact of different sample sizes 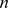 on gene selection strategies with simulation based on biological data. Mean (STD) of **false positives** computed from **SIMU-BIO** with 20 repetitions are reported. Total number of genes: 9005. Number of permutations for Nstat: 100000. The significance threshold: 0.05. **Table S9.** The numbers of differentially expressed genes detected by different selection strategies. Total number of genes: 9005. Number of permutations for Nstat: 100000. The significance threshold: 0.05. **Figure S1.** Histogram of pairwise Pearson correlation coefficients between genes computed from **HYPERDIP** without normalization. Number of genes: 9005. Number of arrays: 88.(PDF)

## References

[1] Tusher VG, Tibshirani R, Chu G (2001) Significance analysis of microarrays applied to the ionizing radiation response. Proc Natl Acad Sci U S A 98: 5116–5121. 11309499 10.1073/pnas.091062498 PMC33173

[2] Sidak Z (1967) Rectangular confidence regions for the means of multivariate normal distributions. Journal of the American Statistical Association 62: 626–633.

[3] Holm S (1979) A simple sequentially rejective multiple test procedure. Scandinavian Journal of Statistics 6: 65–70.

[4] Simes R (1986) An improved bonferroni procedure for multiple tests of significance. Biometrika 73: 751.

[5] Westfall PH, Young SS (1993) Resampling-Based Multiple Testing. Wiley, New York.

[6] Benjamini Y, Hochberg Y (1995) Controlling the false discovery rate: A practical and powerful approach to multiple testing. Journal of the Royal Statistical Society: Series B 57: 289–300.

[7] Dudoit S, Yang YH, Callow MJ, Speed TP (2002) Statistical methods for identifying differentially expressed genes in replicated cdna microarray experiments. Statistica Sinica 12: 111–139.

[8] Lee MLT (2004) Analysis of Microarray Gene Expression Data. Springer, New York.

[9] Bremer M, Himelblau E, Madlung A (2010) Introduction to the statistical analysis of two-color microarray data. Methods Mol Biol 620: 287–313. 20652509 10.1007/978-1-60761-580-4_9

[10] Yakovlev AY, Klebanov L, Gaile D (2010) Statistical Methods for Microarray Data Analysis. Springer, New York.

[11] Hartemink AJ, Gifford DK, Jaakkola TS, Young RA (2001) Maximum likelihood estimation of optimal scaling factors for expression array normalization. SPIE BIOS.

[12] Scherer A (2009) Batch Effects and Noise in Microarray Experiments: Sources and Solutions. Wiley.

[13] Yang YH, Dudoit S, Luu P, Lin DM, Peng V, et al (2002) Normalization for cdna microarray data: a robust composite method addressing single and multiple slide systematic variation. Nucleic Acids Res 30: e15. 11842121 10.1093/nar/30.4.e15 PMC100354

[14] Szabo A, Boucher K, Carroll W, Klebanov L, Tsodikov A, et al (2002) Variable selection and pattern recognition with gene expression data generated by the microarray technology. Mathematical Biosciences 176: 71–98. 11867085 10.1016/s0025-5564(01)00103-1

[15] Tsodikov A, Szabo A, Jones D (2002) Adjustments and measures of differential expression for microarray data. Bioinformatics 18: 251–260. 11847073 10.1093/bioinformatics/18.2.251

[16] Bolstad B, Irizarry R, Astrand M, Speed T (2003) A comparison of normalization methods for high density oligonucleotide array data based on variance and bias. Bioinformatics 19: 185–193. 12538238 10.1093/bioinformatics/19.2.185

[17] Klebanov L, Qiu X, Yakovlev A (2008) Testing differential expression in non-overlapping gene pairs: A new perspective for the empirical Bayes method. Journal of Bioinformatics and Computational Biology 6: 301–316. 18464324 10.1142/s0219720008003436

[18] Klebanov L, Yakovlev A (2008) Diverse correlation structures in gene expression data and their utility in improving statistical inference. Annals of Applied Statistics 1: 538–559.

[19] Quackenbush J (2002) Microarray data normalization and transformation. Nat Genet 32 Suppl: 496–501 10.1038/ng1032 12454644

[20] Bilban M, Buehler LK, Head S, Desoye G, Quaranta V (2002) Normalizing dna microarray data. Curr Issues Mol Biol 4: 57–64. 11931570

[21] Leek JT, Storey JD (2007) Capturing heterogeneity in gene expression studies by surrogate variable analysis. PLoS Genetics 3: e161. 10.1371/journal.pgen.0030161 PMC1994707 17907809

[22] Park T, Yi S, Kang S, Lee S, Lee Y, et al (2003) Evaluation of normalization methods for microarray data. BMC Bioinformatics 4: 33. 12950995 10.1186/1471-2105-4-33 PMC200968

[23] Rao Y, Lee Y, Jarjoura D, Ruppert AS, Liu CG, et al (2008) A comparison of normalization techniques for microrna microarray data. Stat Appl Genet Mol Biol 7: Article22. 18673291 10.2202/1544-6115.1287

[24] Pradervand S, Weber J, Thomas J, Bueno M, Wirapati P, et al (2009) Impact of normalization on mirna microarray expression profiling. RNA 15: 493–501. 19176604 10.1261/rna.1295509 PMC2657010

[25] Qiu X, Wu H, Hu R (2013) The impact of quantile and rank normalization procedures on the testing power of gene differential expression analysis. BMC bioinformatics 14: 124. 23578321 10.1186/1471-2105-14-124 PMC3660216

[26] Yeoh EJ, Ross ME, Shurtleff SA, Williams WK, Patel D, et al (2002) Classification, subtype discovery, and prediction of outcome in pediatric acute lymphoblastic leukemia by gene expression profiling. Cancer Cell 1: 133–143. 12086872 10.1016/s1535-6108(02)00032-6

[27] Qiu X, Brooks AI, Klebanov L, Yakovlev A (2005) The effects of normalization on the correlation structure of microarray data. BMC Bioinformatics 6: 120. 15904488 10.1186/1471-2105-6-120 PMC1156869

[28] Smyth GK (2005) Limma: linear models for microarray data. In: Gentleman R, Carey V, Dudoit S, Irizarry R, Huber W, editors, Bioinformatics and Computational Biology Solutions Using R and Bioconductor, New York: Springer. 397–420.

[29] Szabo A, Boucher K, Jones D, Tsodikov AD, Klebanov LB, et al (2003) Multivariate exploratory tools for microarray data analysis. Biostatistics 4: 555–567. 14557111 10.1093/biostatistics/4.4.555

[30] Xiao Y, Frisina R, Gordon A, Klebanov L, Yakovlev A (2004) Multivariate search for differentially expressed gene combinations. BMC Bioinformatics 5: 164. 15507138 10.1186/1471-2105-5-164 PMC529250

[31] Klebanov L, Gordon A, Xiao Y, Land H, Yakovlev A (2005) A permutation test motivated by microarray data analysis. Computational Statistics and Data Analysis.

[32] Hu R, Qiu X, Glazko G, Klebanov L, Yakovlev A (2009) Detecting intergene correlation changes in microarray analysis: a new approach to gene selection. BMC Bioinformatics 10: 20. 19146700 10.1186/1471-2105-10-20 PMC2657217

[33] Hu R, Qiu X, Glazko G (2010) A new gene selection procedure based on the covariance distance. Bioinformatics 26: 348–354. 19996162 10.1093/bioinformatics/btp672 PMC2815661

[34] McMurray HR, Sampson ER, Compitello G, Kinsey C, Newman L, et al (2008) Synergistic response to oncogenic mutations defines gene class critical to cancer phenotype. Nature 453: 1112–1116. 18500333 10.1038/nature06973 PMC2613942

[35] Dai M, Wang P, Boyd AD, Kostov G, Athey B, et al (2005) Evolving gene/transcript definitions significantly alter the interpretation of GeneChip data. Nucleic Acids Res 33: e175. 16284200 10.1093/nar/gni179 PMC1283542

[36] Ni TT, Lemon WJ, Shyr Y, Zhong TP (2008) Use of normalization methods for analysis of microarrays containing a high degree of gene effects. BMC Bioinformatics 9: 505. 19040742 10.1186/1471-2105-9-505 PMC2612699

[37] Qin LX, Satagopan JM (2009) Normalization method for transcriptional studies of heterogeneous samples–simultaneous array normalization and identification of equivalent expression. Stat Appl Genet Mol Biol 8: Article 10. 10.2202/1544-6115.1339 PMC2861326 19222377

[38] Ogunnaike BA, Gelmi CA, Edwards JS (2010) A probabilistic framework for microarray data analysis: fundamental probability models and statistical inference. J Theor Biol 264: 211–222. 20170665 10.1016/j.jtbi.2010.02.021

[39] Huber W, Von Heydebreck A, Sültmann H, Poustka A, Vingron M (2002) Variance stabilization applied to microarray data calibration and to the quantification of differential expression. Bioinformatics 18: S96–S104. 12169536 10.1093/bioinformatics/18.suppl_1.s96

[40] Lin S, Du P, Huber W, Kibbe W (2008) Model-based variance-stabilizing transformation for illumina microarray data. Nucleic acids research 36: e11. 18178591 10.1093/nar/gkm1075 PMC2241869

[41] Okoniewski M, Miller C (2008) Comprehensive analysis of affymetrix exon arrays using bioconductor. PLoS Comput Biol 4: e6. 18463711 10.1371/journal.pcbi.0040006 PMC2323405

[42] Robinson MD, Speed TP (2007) A comparison of affymetrix gene expression arrays. BMC Bioinformatics 8: 449. 18005448 10.1186/1471-2105-8-449 PMC2216046

[43] Du P, KibbeWA, Lin SM (2008) lumi: a pipeline for processing illumina microarray. Bioinformatics 24: 1547–1548. 18467348 10.1093/bioinformatics/btn224

[44] Schmid R, Baum P, Ittrich C, Fundel-Clemens K, Huber W, et al (2010) Comparison of normalization methods for illumina beadchip humanht-12 v3. BMC Genomics 11: 349. 20525181 10.1186/1471-2164-11-349 PMC3091625

[45] Dunning MJ, Smith ML, Ritchie ME, Tavar S (2007) beadarray: R classes and methods for illumina bead-based data. Bioinformatics 23: 2183–2184. 17586828 10.1093/bioinformatics/btm311

[46] Bullard JH, Purdom E, Hansen KD, Dudoit S (2010) Evaluation of statistical methods for normalization and differential expression in mrna-seq experiments. BMC Bioinformatics 11: 94. 20167110 10.1186/1471-2105-11-94 PMC2838869

[47] Staaf J, Vallon-Christersson J, Lindgren D, Juliusson G, Rosenquist R, et al (2008) Normalization of illumina infinium whole-genome snp data improves copy number estimates and allelic intensity ratios. BMC Bioinformatics 9: 409. 18831757 10.1186/1471-2105-9-409 PMC2572624

[48] 't Hoen P, Ariyurek Y, Thygesen H, Vreugdenhil E, Vossen R, et al.. (2008) Deep sequencing-based expression analysis shows major advances in robustness, resolution and inter-lab portability over five microarray platforms. Nucleic acids research. 10.1093/nar/gkn705 PMC2588528 18927111

[49] Gagnon-Bartsch JA, Speed TP (2012) Using control genes to correct for unwanted variation in microarray data. Biostatistics 13: 539–552. 22101192 10.1093/biostatistics/kxr034 PMC3577104

[50] Johnson WE, Li C, Rabinovic A (2007) Adjusting batch effects in microarray expression data using empirical bayes methods. Biostatistics 8: 118–127. 16632515 10.1093/biostatistics/kxj037

