# Supplementary material for: Evaluation of Bias-Variance Trade-Off for Commonly Used Post-Summarizing Normalization Procedures in Large-Scale Gene Expression Studies
Source: PLoS One. 2014 Jun 18;9(6):e99380. doi: 10.1371/journal.pone.0099380 (PMC4062409; doi:10.1371/journal.pone.0099380)
Supplement: File S1 — Supporting tables and figures. Table S1. The impact of different effect sizes on gene selection strategies when the sample size is fixed and relatively small. Mean (STD) of true positives computed from SIMU1 with 20 repetitions are reported. Sample size: . Total number of genes: 1000. Number of differentially expressed genes: 100. Number of permutations for Nstat: 10000. The significance threshold: 0.05. Table S2. The impact of different effect sizes on gene selection strategies when the sample size is fixed and relatively small. Mean (STD) of false positives computed from SIMU1 with 20 repetitions are reported. Sample size: . Total number of genes: 1000. Number of differentially expressed genes: 100. Number of permutations for Nstat: 10000. The significance threshold: 0.05. Table S3. The impact of different sample sizes on gene selection strategies when the effect size is fixed and relatively small. Mean (STD) of true positives computed from SIMU2 with 20 repetitions are reported. Effect size: . Total number of genes: 1000. Number of differentially expressed genes: 100. Number of permutations for Nstat: 10000. The significance threshold: 0.05. Table S4. The impact of different sample sizes on gene selection strategies when the effect size is fixed and relatively small. Mean (STD) of false positives computed from SIMU2 with 20 repetitions are reported. Effect size: . Total number of genes: 1000. Number of differentially expressed genes: 100. Number of permutations for Nstat: 10000. The significance threshold: 0.05. Table S5. The impact of different sample sizes on gene selection strategies when the effect size is fixed and relatively large. Mean (STD) of true positives computed from SIMU2 with 20 repetitions are reported. Effect size: . Total number of genes: 1000. Number of differentially expressed genes: 100. Number of permutations for Nstat: 10000. The significance threshold: 0.05. Table S6. The impact of different sample sizes on gene selection strategies when t [file pone.0099380.s001.pdf]

# Supplementary Materials for Evaluation of Bias-variance Trade-off for Commonly Used Post-summarizing Normalization Procedures in Large-Scale Gene Expression Studies

Xing Qiu* Rui Hu Zhixin Wu

# 1 The $N$-test

We choose a multivariate nonparametric $N$-distance with the Euclidean kernel as a measure of the distance between two multivariate probability distributions. Using the same notation as in the main text, the sample $N$-distance across conditions $A$ and $B$ for gene $i$ is defined as follows:

$$\begin{aligned} N_i &= \frac{2}{n^2}\sum_{k=1}^{n}\sum_{l=1}^{n} L(x_{ik}^A, x_{il}^B) \\ &\quad -\frac{1}{n^2}\sum_{k=1}^{n}\sum_{l=1}^{n} L(x_{ik}^A, x_{il}^A) \\ &\quad -\frac{1}{n^2}\sum_{k=1}^{n}\sum_{l=1}^{n} L(x_{ik}^B, x_{il}^B), \end{aligned}$$

where $L(x, y) = |x - y|$ is the kernel defined by the Euclidean distance. We apply the following algorithm to calculate the permutation-based $p$-values.

1. Randomly shuffle the arrays in two different conditions, then split them into two groups of equal size.

2. Compute the $N$-statistic for each gene.

3. Repeat the above steps for $K = 100,000$ times, record the permutation based $N$-statistics as $N_{ik}, i = 1, \ldots, m,\ k = 1, \ldots, K$. They can be used to construct the permutation based null distribution for each index $i$.

4. Compute $N_i$, the $N$-statistic for each gene without random shuffles.

5. Obtain the permutation based $p$-value, $p_i$, by comparing $N_i$ with the null distribution constructed from $N_{ik}$. Specifically, $p_i$ is defined to be $\frac{\#(N_{ik} \geqslant N_i)}{K}$, the proportion of $N_{ik}$ which is greater than or equal to $N_i$.

# 2 Analytical Evaluation of Different Normalization Methods Based on the Mixed Effect Model

Based on the assumptions stated in Equation (6) (in Section "The Bias-Variance Trade-off of Normalization Methods") of the main text, we have

$$\mathrm{E}\left(y_{ij}^c\right) = \begin{cases} e^+ & c = A,\ i \in G_1^+, \\ e^- & c = A,\ i \in G_1^-, \\ 0 & c = A,\ i \in G_0, \\ 0 & c = B, \end{cases} \tag{1}$$

## 2.1 Global normalization

According to the definition of global normalization (Equation (1) in the main text), the normalized gene expressions are

$$y_{ij}^{*c} = y_{ij}^c - \bar{y}_{\cdot j}^c = y_{ij}^c - \frac{1}{m}\sum_{k=1}^m y_{kj}^c.$$

Thus

$$\begin{aligned} \mathrm{E}\left(y_{ij}^{*c}\right) &= \mathrm{E}\left(y_{ij}^c - \bar{y}_{\cdot j}^c\right) \\ &= \begin{cases} e^+ - \frac{m_1^+ e^+ + m_1^- e^-}{m}, & c = A,\ i \in G_1^+, \\ e^- - \frac{m_1^+ e^+ + m_1^- e^-}{m}, & c = A,\ i \in G_1^-, \\ -\frac{m_1^+ e^+ + m_1^- e^-}{m}, & c = A,\ i \in G_0, \\ 0, & c = B. \end{cases} \end{aligned} \tag{2}$$

By comparing Equation (2) with Equation (1), it is clear that the global normalization introduces a small bias $-\mathrm{E}\bar{y}_{\cdot j}^c = -\frac{m_1^+ e^+ + m_1^- e^-}{m}$. More specifically, the expected mean group difference for global normalized data are

$$\mathrm{E}\left(y_{ij}^{*A} - y_{ij}^{*B}\right) = \begin{cases} e^+ - \frac{m_1^+ e^+ + m_1^- e^-}{m}, & i \in G_1^+, \\ e^- - \frac{m_1^+ e^+ + m_1^- e^-}{m}, & i \in G_1^-, \\ -\frac{m_1^+ e^+ + m_1^- e^-}{m}, & i \in G_0. \end{cases} \tag{3}$$

The variances of global normalized gene expressions are

$$\begin{aligned} \mathrm{var}(y_{ij}^{*c}) &= \mathrm{var}\left(\frac{m-1}{m} y_{ij}^c - \frac{1}{m}\sum_{k \neq i} y_{kj}^c\right) \\ &= \frac{(m-1)^2}{m^2}\sigma_{ic}^2 + \frac{1}{m^2}\sum_{k\neq i}\sum_{l \neq i}\sigma_{kc}\sigma_{lc}\tilde{\rho}_{kl}^c \\ &\quad - \frac{2(m-1)}{m^2}\sum_{k \neq i}\sigma_{kc}\sigma_{ic}\tilde{\rho}_{ik}^c. \end{aligned} \tag{4}$$

## 2.2 Quantile normalization

We investigate the bias effects through the following two aspects.

1. The rank skewing effect. Over(under)-expressed genes tend to have very high (low) ranks. It means that the NDEGs are much more likely to take only medium ranks. When the effect size is large, the DEGs in group $A$ effectively take up all the top and bottom ranks, so the NDEGs in group $A$ can only compete for ranks between $m_1^- + 1$ and $m - m_1^+$. For simplicity, we assume all NDEGs in group $A$ have equal chances to take ranks from $m_1^- + 1$ to $m - m_1^+$. The expectation of $y_{i\cdot}^{*A}$, $i \in G_0$, is

$$
\begin{aligned}
\mathrm{E}\left(y_{i\cdot}^{*A}\right) &= \mathrm{E}(\mathrm{E}(y_{i\cdot}^{*A}|r_{i\cdot}^{A})) \approx \frac{1}{m_0} \sum_{r=m_1^-+1}^{m-m_1^+} \mathrm{E}\left(q_r\right) \\
&\approx \frac{1}{2m_0 n} \left( \sum_{k=1}^{n} \sum_{j \in G_0} \mathrm{E}(y_{jk}^{A}) + \sum_{l=1}^{n} \sum_{r=m_1^-+1}^{m-m_1^+} \mathrm{E}(y_{(r),l}^{B}) \right) \\
&= \frac{n}{2m_0 n} \left( \sum_{r=1}^{m} \mathrm{E}(y_{(r),\cdot}^{B}) - \sum_{r=1}^{m_1^-} \mathrm{E}(y_{(r),\cdot}^{B}) - \sum_{r=m_1^-+m_0+1}^{m} \mathrm{E}(y_{(r),\cdot}^{B}) \right) \\
&= \frac{1}{2m_0} \left( \mathrm{E}\left( \sum_{j=1}^{m} y_{j\cdot}^{B} \right) - \sum_{r=1}^{m_1^-} \mathrm{E}(y_{(r),\cdot}^{B}) - \sum_{r=m_1^-+m_0+1}^{m} \mathrm{E}(y_{(r),\cdot}^{B}) \right) \\
&= -\frac{m_1^+ \delta_1 + m_1^- \delta_2}{2m_0},
\end{aligned}
$$

where $\delta_1 = \frac{1}{m_1^+} \sum_{r=m_1^-+m_0+1}^{m} \mathrm{E}(y_{(r),\cdot}^{B})$ and $\delta_2 = \frac{1}{m_1^-} \sum_{r=1}^{m_1^-} \mathrm{E}(y_{(r),\cdot}^{B})$. $\delta_1$ represents the mean expectation of the top $m_1^+$ ordered expression levels and $\delta_2$ represents the mean expectation of the bottom $m_1^-$ ordered expression levels, provided that there is no differentially expressed gene. Clearly $\delta_1$ and $\delta_2$ do not depend on $e^+$ and $e^-$. We assume all genes in group $B$ have equal chances to take ranks from 1 to $m$. For $i = 1, \ldots, m$,

$$
\begin{aligned}
\mathrm{E}\left(y_{i\cdot}^{*B}\right) &= \mathrm{E}\left(\mathrm{E}(y_{i\cdot}^{*B}|r_{i\cdot}^{B})\right) = \frac{1}{m} \mathrm{E}\left( \sum_{r=1}^{m} q_r \right) \\
&= \frac{1}{2nm} \mathrm{E}\left( \sum_{k=1}^{n} \sum_{r=1}^{m} y_{(r),k}^{A} + \sum_{l=1}^{n} \sum_{r=1}^{m} y_{(r),l}^{B} \right) \\
&= \frac{1}{2nm} \left( \sum_{k=1}^{n} \sum_{j=1}^{m} \mathrm{E}(y_{jk}^{A}) + \sum_{l=1}^{n} \sum_{j=1}^{m} \mathrm{E}(y_{jl}^{B}) \right) \\
&= \frac{m_1^+ e^+ + m_1^- e^-}{2m}.
\end{aligned}
$$

The difference between these two expectations explains the spurious effect introduced to NDEGs by the quantile normalization. A similar argument also explains the bias introduced by the rank normalization, which will be discussed later.

2. The averaging effect. Denote the reference quantile array constructed from one group by $\mathbf{q}^c$, $c = A, B$, we have

$$
q_r = \frac{q_r^A + q_r^B}{2}, \quad q_r^c = \frac{1}{n} \sum_{k=1}^{n} y_{(r),k}^{c}, \; c = A, B. \tag{5}
$$

In other words, the reference array $\mathbf{q}$ is computed by averaging both DEGs and NDEGs over arrays in two phenotypic groups, so the $m_1^+$ over-expressed genes and $m_1^-$ under-expressed genes in group $A$ are "mixed up" with NDEGs of the same rank from group $B$. In fact, assuming that the $m_1^+$ up-regulated

genes almost always take the top $m_1^+$ ranks with equal chances and the $m_1^-$ down-regulated genes almost always take the bottom $m_1^-$ ranks with equal chances, we can compute that for $i \in G_1^+$,

$$\begin{aligned}
\mathrm{E}\left(y_{i\cdot}^{*A}\right) &\approx \frac{1}{m_1^+} \sum_{r=m_1^-+m_0+1}^{m} \mathrm{E}\left(q_r\right) \\
&\approx \frac{1}{2m_1^+ n} \left( \sum_{k=1}^{n} \sum_{j \in G_1^+} \mathrm{E}(y_{jk}^A) + \sum_{l=1}^{n} \sum_{r=m_1^-+m_0+1}^{m} \mathrm{E}(y_{(r),l}^B) \right) \\
&= \frac{e^+ + \delta_1}{2}.
\end{aligned}$$

and for $i \in G_1^-$,

$$\begin{aligned}
\mathrm{E}\left(y_{i\cdot}^{*A}\right) &\approx \frac{1}{m_1^-} \sum_{r=1}^{m_1^-} \mathrm{E}\left(q_r\right) \\
&\approx \frac{1}{2m_1^- n} \left( \sum_{k=1}^{n} \sum_{j \in G_1^-} \mathrm{E}(y_{jk}^A) + \sum_{l=1}^{n} \sum_{r=1}^{m_1^-} \mathrm{E}(y_{(r),l}^B) \right) \\
&= \frac{e^- + \delta_2}{2}.
\end{aligned}$$

As mentioned before, we focus on the case in which the up-regulated (down-regulated) genes almost always take the top (bottom) ranks. Recall that $\delta_1$ ($\delta_2$) represents the mean expression of the top $m_1^+$ (bottom $m_1^-$) genes, given that there is no DEG. In order for up-regulated (down-regulated) genes to almost always take up the top $m_1^+$ (bottom $m_1^-$) ranks, we must have

$$e^+ \gg \delta_1, \quad e^- \ll \delta_2. \tag{6}$$

Thus $|\mathrm{E}(y_{i\cdot}^{*A})|$ is smaller than the original effect size.

Based on the above calculations, the expected group differences are

$$\begin{aligned}
&\mathrm{E}\left(y_{i\cdot}^{*A} - y_{i\cdot}^{*B}\right) \\
&\approx \begin{cases}
\frac{e^+}{2} - \frac{m_1^+ e^+ + m_1^- e^-}{2m} + \frac{\delta_1}{2}, & i \in G_1^+, \\
\frac{e^-}{2} - \frac{m_1^+ e^+ + m_1^- e^-}{2m} + \frac{\delta_2}{2}, & i \in G_1^-, \\
-\frac{m_1^+ \delta_1 + m_1^- \delta_2}{2m_0} - \frac{m_1^+ e^+ + m_1^- e^-}{2m}, & i \in G_0.
\end{cases}
\end{aligned} \tag{7}$$

## 2.3 Rank normalization

The number of genes ($m$) is usually very large in a typical microarray study. If the effect size is large such that the over-expressed genes always take up the top $m_1^+$ ranks and the under-expressed genes always take up the bottom $m_1^-$ ranks in group $A$, $y_{ij}^{*c}$ approximately has the following uniform distribution:

$$y_{ij}^{*c} \sim \begin{cases}
U(1 - \frac{m_1^+}{m}, 1), & c = A,\ i \in G_1^+, \\
U(0, \frac{m_1^-}{m}), & c = A,\ i \in G_1^-, \\
U(\frac{m_1^-}{m}, 1 - \frac{m_1^+}{m}), & c = A,\ i \in G_0, \\
U(0, 1), & c = B.
\end{cases} \tag{8}$$

Here we assume that the genes take the specified ranks with equal chances for simplicity.

The expected group expression differences are

$$\mathrm{E}\left(y_{i\cdot}^{*A} - y_{i\cdot}^{*B}\right) \approx \begin{cases} \frac{1}{2} - \frac{m_1^+}{2m} & i \in G_1^+, \\ \frac{m_1^-}{2m} - \frac{1}{2} & i \in G_1^-, \\ -\frac{m_1^+ + m_1^-}{2m} & i \in G_0. \end{cases} \tag{9}$$

## 2.4 $\delta$-sequence method

The variance reduction effect of the $\delta$-sequence method comes from the gene pairing and subtraction. From Equation (5) in the main text, we have

$$\begin{aligned} & \mathrm{var}(d_{kj}^{*c}) \\ = \; & \mathrm{var}(y_{o_{2k-1}j}^c - y_{o_{2k}j}^c) \\ = \; & \mathrm{var}(y_{o_{2k-1}j}^c) + \mathrm{var}(y_{o_{2k}j}^c) - 2\,\mathrm{cov}\left(y_{o_{2k-1}j}^c, \, y_{o_{2k}j}^c\right) \\ = \; & \sigma_{o_{2k-1},c}^2 + \sigma_{o_{2k},c}^2 - 2\tilde{\rho}_{o_{2k-1},o_{2k}} \sigma_{o_{2k-1},c} \sigma_{o_{2k},c}. \end{aligned}$$

Thanks to sorting by sample variance, we have $\sigma_{o_{2k-1},c}^2 \approx \sigma_{o_{2k},c}^2$. If we plug in $\tilde{\rho}_{o_{2k-1},o_{2k}} = 0.9$ for all $k$ as used in our simulations, we have $\mathrm{var}(d_{kj}^{*c}) \approx 0.2\sigma_{o_{2k-1},c}^2$.

# 3 Results of simulation studies

From Tables S1, S3 and S5, we see that gene selection strategies based on both $t$-test and $N$-test almost always have comparable or better statistical power than the Wilcoxon rank-sum test for **SIMU1**, **SIMU2**, and **SIMU3**. The comparison between the $t$-test and $N$-test are not that clear cut, and the pattern seems to be:

1. The $t$-test has comparable or better statistical power than the $N$-test with **NONE**, **GLOBAL**, **QUANT** and **DELTA**.
2. The $N$-test outperforms $t$-test with **RANK**.

It is well known that the Student's $t$-test is optimal when the inputs (normalized data) are normally distributed. In this study, **SIMU1**, **SIMU2**, and **SIMU3** are all simulated based on Gaussian models. Furthermore, both **GLOBAL** and **DELTA** are *linear* transformations of data hence they preserve normality. **QUANT** transforms data by using quantiles of mixture normal distribution, so it also preserves normality to a certain extent. Hence it is no coincidence that the $t$-test attains best power in these situations. On the other hand, rank normalized data are highly non-normal. The $N$-test outperforms the $t$-test in this situation because it is a nonparametric test which is sensitive to a large class of distributional differences.

For **SIMU-BIO** S7 we observe some interesting differences. When applied to the normalized data, the $N$-test (or even the Wilcoxon rank-sum test) outperforms the $t$-test most of the time. The $N$-test also outperforms the $t$-test with unbalanced differential structure and a large sample size ($n = 79$) when no normalization is applied. This suggests that the original and normalized gene expressions in the biological data may not be normally distributed.

From Tables S1, S3, S5 and S7, we see that gene selection strategies with **BH** have better statistical testing power compared with those with **BONF**. From Tables S2, S4, S6 and S8, we see that **BH** allows for more false positives for normalized data than **BONF**. It is consistent with the known fact that **BONF** is more conservative than **BH** given the same significance level. We also observe that for the non-normalized data, **BH** is more unstable compared with **BONF** in terms of the variances of the numbers of true and false positives.

Table S1: The impact of different effect sizes $e$ on gene selection strategies when the sample size $n$ is fixed and relatively small. Mean (STD) of **true positives** computed from **SIMU1** with 20 repetitions are reported. Sample size: $n = 10$. Total number of genes: 1000. Number of differentially expressed genes: 100. Number of permutations for Nstat: 10000. The significance threshold: 0.05.

| | | | **UP 60, DOWN 40** | | | **UP 90, DOWN 10** | | |
|---|---|---|---|---|---|---|---|---|
| Norm | Test | MTP | e=0.2 | e=1.0 | e=1.8 | e=0.2 | e=1.0 | e=1.8 |
| NONE | t | BONF | 0(0) | 84.15(21.25) | 100(0) | 0(0) | 87.5(19.01) | 100(0) |
| NONE | t | BH | 0(0) | 99.4(2.68) | 100(0) | 0(0) | 99.85(0.67) | 100(0) |
| NONE | LIMMA | BONF | 0(0) | 95.45(14.46) | 100(0) | 0(0) | 98.05(7.6) | 100(0) |
| NONE | LIMMA | BH | 0(0) | 99.95(0.22) | 100(0) | 0(0) | 100(0) | 100(0) |
| NONE | Wilcox | BONF | 0(0) | 65.95(28.4) | 99.9(0.45) | 0(0) | 68.65(31.64) | 99.85(0.67) |
| NONE | Wilcox | BH | 0(0) | 99.15(3.8) | 100(0) | 0(0) | 99.75(1.12) | 100(0) |
| NONE | Nstat | BONF | 0(0) | 80.55(22.43) | 99.65(1.57) | 0(0) | 82.45(25.36) | 99.45(2.46) |
| NONE | Nstat | BH | 0(0) | 99.55(2.01) | 100(0) | 0(0) | 99.85(0.67) | 100(0) |
| GLOBAL | t | BONF | 19.7(3.6) | 100(0) | 100(0) | 15.5(2.87) | 100(0) | 100(0) |
| GLOBAL | t | BH | 73.9(4.75) | 100(0) | 100(0) | 64.05(5.4) | 100(0) | 100(0) |
| GLOBAL | LIMMA | BONF | 48.55(4.54) | 100(0) | 100(0) | 39(3.95) | 100(0) | 100(0) |
| GLOBAL | LIMMA | BH | 87.65(3.45) | 100(0) | 100(0) | 84.15(2.94) | 100(0) | 100(0) |
| GLOBAL | Wilcox | BONF | 14.5(3.86) | 100(0) | 100(0) | 10.75(2.36) | 100(0) | 100(0) |
| GLOBAL | Wilcox | BH | 67.55(5.6) | 100(0) | 100(0) | 59.15(6.12) | 100(0) | 100(0) |
| GLOBAL | Nstat | BONF | 20.2(4.55) | 100(0) | 100(0) | 16.45(3.17) | 100(0) | 100(0) |
| GLOBAL | Nstat | BH | 70.85(5.15) | 100(0) | 100(0) | 62.75(4.85) | 100(0) | 100(0) |
| QUANT | t | BONF | 16.2(3.94) | 94.3(2.56) | 95.05(2.67) | 12.25(2.73) | 89.85(3.47) | 92.25(2) |
| QUANT | t | BH | 63.75(6.02) | 100(0) | 99.95(0.22) | 55.55(5.16) | 99.75(0.55) | 99.9(0.31) |
| QUANT | LIMMA | BONF | 35(4.39) | 98(1.62) | 98.05(1.15) | 26.3(3.4) | 95.45(1.79) | 95.6(2.01) |
| QUANT | LIMMA | BH | 77.8(4.19) | 99.95(0.22) | 100(0) | 71.55(3.5) | 99.95(0.22) | 99.9(0.31) |
| QUANT | Wilcox | BONF | 10.95(3.27) | 69.9(4.58) | 67.7(4.52) | 8.7(2.39) | 56.15(4.3) | 56.25(5.04) |
| QUANT | Wilcox | BH | 57.45(5.24) | 97.75(1.8) | 97.45(1.82) | 48(6.24) | 94.55(2.8) | 93(2.34) |
| QUANT | Nstat | BONF | 16.75(4.13) | 84.7(3.29) | 82.8(3.3) | 13.85(3.48) | 74.75(4.6) | 72.55(3.71) |
| QUANT | Nstat | BH | 62.25(6.27) | 100(0) | 99.9(0.45) | 53.9(5.15) | 99.6(0.68) | 99.45(0.6) |
| RANK | t | BONF | 15.75(2.81) | 46.4(5.38) | 46.1(3.97) | 13.05(3.46) | 41.8(3.98) | 40.65(4.42) |
| RANK | t | BH | 60.65(6.34) | 97.8(1.64) | 97.85(1.42) | 53.65(4.82) | 96.6(2.04) | 96.4(1.54) |
| RANK | LIMMA | BONF | 12.5(3.05) | 40.75(4.02) | 42.3(4.74) | 9.4(3.17) | 37.95(4.36) | 36.7(4.26) |
| RANK | LIMMA | BH | 55.65(6.16) | 92.1(2.45) | 91.65(2.74) | 49(6.22) | 90.05(3.19) | 89.6(2.28) |
| RANK | Wilcox | BONF | 10.95(3.27) | 69.9(4.58) | 67.7(4.52) | 8.7(2.39) | 56.15(4.3) | 56.25(5.04) |
| RANK | Wilcox | BH | 57.45(5.24) | 97.75(1.8) | 97.45(1.82) | 48(6.24) | 94.55(2.8) | 93(2.34) |
| RANK | Nstat | BONF | 16.6(3.72) | 83.65(3.51) | 82.25(3.54) | 13.9(3.13) | 73.9(4.4) | 71.7(3.51) |
| RANK | Nstat | BH | 58.8(6.83) | 100(0) | 99.85(0.49) | 52.45(4.67) | 99.55(0.69) | 99.3(0.66) |
| DELTA | t | BONF | 2.4(1.5) | 89.95(3.89) | 91.55(3.83) | 1.85(1.63) | 85(4.4) | 86.45(3.97) |
| DELTA | t | BH | 20.4(5.84) | 89.95(3.89) | 91.75(3.91) | 18.65(4.32) | 85.05(4.44) | 86.65(4.09) |
| DELTA | LIMMA | BONF | 7.7(2.52) | 89.45(3.58) | 90.65(3.28) | 5.35(2.48) | 83.8(6.28) | 84.3(3.59) |
| DELTA | LIMMA | BH | 37.8(5.09) | 89.65(3.75) | 90.65(3.28) | 33.65(5.09) | 83.8(6.28) | 84.5(3.47) |
| DELTA | Wilcox | BONF | 1.5(1.19) | 89.95(3.89) | 91.55(3.83) | 0.95(1) | 85(4.4) | 86.45(3.97) |
| DELTA | Wilcox | BH | 17.3(4.92) | 89.95(3.89) | 91.75(3.91) | 15.1(3.6) | 85.05(4.44) | 86.65(4.09) |
| DELTA | Nstat | BONF | 3.2(1.54) | 89.95(3.89) | 91.55(3.83) | 2.25(1.48) | 85(4.4) | 86.45(3.97) |
| DELTA | Nstat | BH | 19.45(5.34) | 89.95(3.89) | 91.75(3.91) | 17.25(3.27) | 85.05(4.44) | 86.65(4.09) |
| SVA | t | BONF | 21.2(14.12) | 90.7(19.49) | 100(0) | 16.4(17.37) | 95.15(19.67) | 100(0) |
| SVA | t | BH | 62.15(11.77) | 99.55(2.01) | 100(0) | 58.1(24.07) | 100(0) | 100(0) |
| SVA | LIMMA | BONF | 44.6(11.57) | 95.45(14.46) | 100(0) | 37.7(21.58) | 98.1(7.61) | 100(0) |
| SVA | LIMMA | BH | 74.95(14.71) | 99.95(0.22) | 100(0) | 72.25(26.92) | 100(0) | 100(0) |

Table S2: The impact of different effect sizes $e$ on gene selection strategies when the sample size $n$ is fixed and relatively small. Mean (STD) of **false positives** computed from **SIMU1** with 20 repetitions are reported. Sample size: $n = 10$. Total number of genes: 1000. Number of differentially expressed genes: 100. Number of permutations for Nstat: 10000. The significance threshold: 0.05.

| | | | | **UP 60, DOWN 40** | | | **UP 90, DOWN 10** | |
|---|---|---|---|---|---|---|---|---|
| Norm | Test | MTP | e=0.2 | e=1.0 | e=1.8 | e=0.2 | e=1.0 | e=1.8 |
| NONE | t | BONF | 0(0) | 0(0) | 0(0) | 0(0) | 0(0) | 0(0) |
| NONE | t | BH | 0(0) | 0.1(0.31) | 45.2(199.33) | 0(0) | 0.1(0.31) | 45.2(199.33) |
| NONE | LIMMA | BONF | 0(0) | 0(0) | 0(0) | 0(0) | 0(0) | 1.35(6.04) |
| NONE | LIMMA | BH | 0(0) | 1.25(4.91) | 0(0) | 0(0) | 0.35(1.09) | 86.45(266.17) |
| NONE | Wilcox | BONF | 0(0) | 0(0) | 0.1(0.45) | 0(0) | 0(0) | 0.1(0.45) |
| NONE | Wilcox | BH | 0(0) | 0(0) | 43.05(187.13) | 0(0) | 0(0) | 43.05(187.13) |
| NONE | Nstat | BONF | 0(0) | 0(0) | 0.2(0.89) | 0(0) | 0(0) | 0.2(0.89) |
| NONE | Nstat | BH | 0(0) | 0.1(0.45) | 43.1(190.63) | 0(0) | 0.1(0.45) | 43.1(190.63) |
| GLOBAL | t | BONF | 0.05(0.22) | 0.1(0.31) | 0(0) | 0.05(0.22) | 2.75(1.68) | 36.3(6.2) |
| GLOBAL | t | BH | 3.5(1.57) | 7.35(2.74) | 16.2(3.85) | 4.35(1.84) | 132.6(13.74) | 672.3(12.83) |
| GLOBAL | LIMMA | BONF | 0(0) | 0.15(0.37) | 0.45(0.6) | 0.1(0.31) | 5.85(2.43) | 106.4(9.69) |
| GLOBAL | LIMMA | BH | 3.5(2.37) | 8.15(3.08) | 19.3(4.34) | 6.25(2.31) | 183.6(10.54) | 725.75(10.08) |
| GLOBAL | Wilcox | BONF | 0(0) | 0.1(0.31) | 0.05(0.22) | 0(0) | 1.75(1.16) | 25.05(3.63) |
| GLOBAL | Wilcox | BH | 2.95(1.61) | 6.75(2.55) | 14.6(3.49) | 3.6(1.79) | 119.7(17.47) | 641.5(10.97) |
| GLOBAL | Nstat | BONF | 0.15(0.37) | 0.15(0.37) | 0.1(0.31) | 0.25(0.44) | 4.65(2.41) | 48(5.85) |
| GLOBAL | Nstat | BH | 4.05(2.19) | 7.2(3.05) | 16.5(3.72) | 4.6(1.76) | 126.35(12.9) | 645.7(15.31) |
| QUANT | t | BONF | 0.05(0.22) | 0(0) | 0(0) | 0.05(0.22) | 0(0) | 0(0) |
| QUANT | t | BH | 2.95(1.47) | 1.95(1.32) | 1.65(1.27) | 3.45(1.7) | 2.85(1.39) | 2.15(1.27) |
| QUANT | LIMMA | BONF | 0(0) | 0(0) | 0(0) | 0.1(0.31) | 0(0) | 0(0) |
| QUANT | LIMMA | BH | 3.3(2.47) | 1.3(1.03) | 0.7(0.73) | 5.35(2.3) | 3(1.52) | 0.55(0.76) |
| QUANT | Wilcox | BONF | 0(0) | 0.05(0.22) | 0(0) | 0(0) | 0.15(0.37) | 0(0) |
| QUANT | Wilcox | BH | 2.85(1.57) | 3.75(2.34) | 4.45(2.5) | 2.85(1.39) | 4.8(2.57) | 4.85(1.76) |
| QUANT | Nstat | BONF | 0.1(0.31) | 0.1(0.31) | 0.05(0.22) | 0.15(0.37) | 0.15(0.37) | 0(0) |
| QUANT | Nstat | BH | 3.6(1.82) | 4.7(2.3) | 5.2(1.94) | 3.5(1.91) | 6.45(3.19) | 6.35(1.63) |
| RANK | t | BONF | 0.1(0.31) | 0.15(0.37) | 0.05(0.22) | 0.15(0.37) | 0.25(0.44) | 0.05(0.22) |
| RANK | t | BH | 3.85(2.13) | 5.55(2.63) | 5.35(2.08) | 4.35(1.9) | 6.9(2.63) | 7.85(3.01) |
| RANK | LIMMA | BONF | 0(0) | 0(0) | 0(0) | 0.15(0.37) | 0.05(0.22) | 0.05(0.22) |
| RANK | LIMMA | BH | 1.85(1.5) | 4.15(2.01) | 4.8(2.75) | 3.6(1.88) | 6.3(2.34) | 6.35(2.56) |
| RANK | Wilcox | BONF | 0(0) | 0.05(0.22) | 0(0) | 0(0) | 0.15(0.37) | 0(0) |
| RANK | Wilcox | BH | 2.85(1.57) | 3.75(2.34) | 4.45(2.5) | 2.85(1.39) | 4.8(2.57) | 4.85(1.76) |
| RANK | Nstat | BONF | 0.1(0.31) | 0.15(0.37) | 0.05(0.22) | 0.15(0.37) | 0.15(0.37) | 0(0) |
| RANK | Nstat | BH | 3.25(1.71) | 4.15(2.06) | 4.8(1.58) | 3.4(1.82) | 5.3(2.56) | 5.9(2.34) |
| DELTA | t | BONF | 0.15(0.37) | 8.6(3.22) | 9.35(2.64) | 0.15(0.37) | 8.6(3.22) | 9.35(2.64) |
| DELTA | t | BH | 2(1.34) | 11.35(3.54) | 11.9(2.57) | 2.35(1.53) | 11.3(3.48) | 11.9(2.57) |
| DELTA | LIMMA | BONF | 0.25(0.44) | 8.3(2.32) | 9.85(2.25) | 0.6(0.82) | 9.6(3.02) | 9.75(2.92) |
| DELTA | LIMMA | BH | 3.1(1.83) | 10.55(2.21) | 12.3(2.77) | 4.1(2.31) | 12(3.57) | 11.85(3.05) |
| DELTA | Wilcox | BONF | 0.1(0.31) | 8.6(3.22) | 9.35(2.64) | 0(0) | 8.6(3.22) | 9.35(2.64) |
| DELTA | Wilcox | BH | 1.6(1.19) | 11.05(3.27) | 11.9(2.95) | 1.95(1.43) | 11.05(3.27) | 11.85(3.01) |
| DELTA | Nstat | BONF | 0.25(0.44) | 8.6(3.22) | 9.35(2.64) | 0.15(0.37) | 8.6(3.22) | 9.35(2.64) |
| DELTA | Nstat | BH | 2(1.59) | 11.75(3.27) | 12.25(3.09) | 2.25(1.41) | 11.7(3.37) | 12.2(3.04) |
| SVA | t | BONF | 10.8(23.75) | 0.3(0.57) | 0.05(0.22) | 22.4(49.11) | 0.2(0.52) | 0.1(0.45) |
| SVA | t | BH | 159.8(300.59) | 14.55(17.43) | 10.35(13.82) | 172.1(341.4) | 15.1(42.03) | 86.3(255.33) |
| SVA | LIMMA | BONF | 40(91.46) | 0.5(1.1) | 0.1(0.31) | 66.1(142.48) | 0.55(2.24) | 1.45(6.03) |
| SVA | LIMMA | BH | 177.7(317.81) | 22.15(28.05) | 13.1(18.57) | 180.7(350.07) | 22.25(60.51) | 90.5(264.84) |

Table S3: The impact of different sample sizes $n$ on gene selection strategies when the effect size $e$ is fixed and relatively small. Mean (STD) of **true positives** computed from **SIMU2** with 20 repetitions are reported. Effect size: $e = 0.2$. Total number of genes: 1000. Number of differentially expressed genes: 100. Number of permutations for Nstat: 10000. The significance threshold: 0.05.

| | | | **UP 60, DOWN 40** | | | | **UP 90, DOWN 10** | | | |
|---|---|---|---|---|---|---|---|---|---|---|
| Norm | Test | MTP | n=5 | n=10 | n=70 | n=100 | n=5 | n=10 | n=70 | n=100 |
| NONE | t | BONF | 0(0) | 0(0) | 21.9(20.74) | 44.55(13.18) | 0(0) | 0(0) | 23.1(31.31) | 41.6(33.45) |
| NONE | t | BH | 0(0) | 0(0) | 62.45(17.22) | 91.4(13.28) | 0(0) | 0(0) | 56.25(42.28) | 92.7(19.23) |
| NONE | LIMMA | BONF | 0(0) | 0(0) | 27.5(20.64) | 53.6(13.15) | 0.05(0.22) | 0(0) | 36.9(37.2) | 48.65(37.53) |
| NONE | LIMMA | BH | 0(0) | 0(0) | 67.4(21.14) | 86.4(17.64) | 0.05(0.22) | 0(0) | 72.9(34.04) | 78.75(35.21) |
| NONE | Wilcox | BONF | 0(0) | 0(0) | 20.8(20.1) | 42.15(13.88) | 0(0) | 0(0) | 21(30.6) | 39.25(34.1) |
| NONE | Wilcox | BH | 0(0) | 0(0) | 50.95(22.94) | 87.75(14.32) | 0(0) | 0(0) | 46.9(43.02) | 87.25(22.48) |
| NONE | Nstat | BONF | 0(0) | 0(0) | 22.75(18.61) | 43.75(14.49) | 0(0) | 0(0) | 24(30.29) | 41.3(34.24) |
| NONE | Nstat | BH | 0(0) | 0(0) | 50.25(21.09) | 87.05(14.08) | 0(0) | 0(0) | 47.1(41.91) | 85.05(25.96) |
| GLOBAL | t | BONF | 0.8(0.77) | 19.7(3.6) | 100(0) | 100(0) | 1(1.08) | 15.5(2.87) | 100(0) | 100(0) |
| GLOBAL | t | BH | 3.9(3.52) | 73.9(4.75) | 100(0) | 100(0) | 3.1(2.9) | 64.05(5.4) | 100(0) | 100(0) |
| GLOBAL | LIMMA | BONF | 10.8(2.98) | 48.55(4.54) | 100(0) | 100(0) | 8.4(2.95) | 39(3.95) | 100(0) | 100(0) |
| GLOBAL | LIMMA | BH | 39(6.2) | 87.65(3.45) | 100(0) | 100(0) | 33.25(8.42) | 84.15(2.94) | 100(0) | 100(0) |
| GLOBAL | Wilcox | BONF | 0(0) | 14.5(3.86) | 100(0) | 100(0) | 0(0) | 10.75(2.36) | 100(0) | 100(0) |
| GLOBAL | Wilcox | BH | 0(0) | 67.55(5.6) | 100(0) | 100(0) | 0(0) | 59.15(6.12) | 100(0) | 100(0) |
| GLOBAL | Nstat | BONF | 0(0) | 20.2(4.55) | 100(0) | 100(0) | 0(0) | 16.45(3.17) | 100(0) | 100(0) |
| GLOBAL | Nstat | BH | 0(0) | 70.85(5.15) | 100(0) | 100(0) | 0(0) | 62.75(4.85) | 100(0) | 100(0) |
| QUANT | t | BONF | 0.6(0.82) | 16.2(3.94) | 100(0) | 100(0) | 0.65(0.81) | 12.25(2.73) | 100(0) | 100(0) |
| QUANT | t | BH | 2.45(2.7) | 63.75(6.02) | 100(0) | 100(0) | 2.5(2.46) | 55.55(5.16) | 100(0) | 100(0) |
| QUANT | LIMMA | BONF | 6.25(2.29) | 35(4.39) | 100(0) | 100(0) | 3.75(2.17) | 26.3(3.4) | 100(0) | 100(0) |
| QUANT | LIMMA | BH | 27.45(5.46) | 77.8(4.19) | 100(0) | 100(0) | 19.55(7.26) | 71.55(3.5) | 100(0) | 100(0) |
| QUANT | Wilcox | BONF | 0(0) | 10.95(3.27) | 100(0) | 100(0) | 0(0) | 8.7(2.39) | 100(0) | 100(0) |
| QUANT | Wilcox | BH | 0(0) | 57.45(5.24) | 100(0) | 100(0) | 0(0) | 48(6.24) | 100(0) | 100(0) |
| QUANT | Nstat | BONF | 0(0) | 16.75(4.13) | 100(0) | 100(0) | 0(0) | 13.85(3.48) | 99.95(0.22) | 100(0) |
| QUANT | Nstat | BH | 0(0) | 62.25(6.27) | 100(0) | 100(0) | 0(0) | 53.9(5.15) | 100(0) | 100(0) |
| RANK | t | BONF | 2(1.62) | 15.75(2.81) | 100(0) | 100(0) | 2.15(1.27) | 13.05(3.46) | 100(0) | 100(0) |
| RANK | t | BH | 6.65(3.87) | 60.65(6.34) | 100(0) | 100(0) | 5.2(2.95) | 53.65(4.82) | 100(0) | 100(0) |
| RANK | LIMMA | BONF | 0.5(0.69) | 12.5(3.05) | 100(0) | 100(0) | 0.7(0.8) | 9.4(3.17) | 100(0) | 100(0) |
| RANK | LIMMA | BH | 2.9(3.6) | 55.65(6.16) | 100(0) | 100(0) | 1.6(2.48) | 49(6.22) | 100(0) | 100(0) |
| RANK | Wilcox | BONF | 0(0) | 10.95(3.27) | 100(0) | 100(0) | 0(0) | 8.7(2.39) | 100(0) | 100(0) |
| RANK | Wilcox | BH | 0(0) | 57.45(5.24) | 100(0) | 100(0) | 0(0) | 48(6.24) | 100(0) | 100(0) |
| RANK | Nstat | BONF | 0(0) | 16.6(3.72) | 100(0) | 100(0) | 0(0) | 13.9(3.13) | 100(0) | 100(0) |
| RANK | Nstat | BH | 0(0) | 58.8(6.83) | 100(0) | 100(0) | 0(0) | 52.45(4.67) | 100(0) | 100(0) |
| DELTA | t | BONF | 0(0) | 2.4(1.5) | 90.75(3.55) | 89.75(4.45) | 0.05(0.22) | 1.85(1.63) | 84.45(5.71) | 83.7(4.75) |
| DELTA | t | BH | 0.45(1.05) | 20.4(5.84) | 90.9(3.61) | 89.75(4.45) | 0.1(0.31) | 18.65(4.32) | 84.8(5.63) | 83.9(4.67) |
| DELTA | LIMMA | BONF | 0.8(0.95) | 7.7(2.52) | 90.75(3.16) | 89.5(4.63) | 0.55(0.6) | 5.35(2.48) | 84.9(3.16) | 85.1(5.07) |
| DELTA | LIMMA | BH | 5.65(2.37) | 37.8(5.09) | 90.9(3.14) | 89.5(4.63) | 5.2(3.16) | 33.65(5.09) | 84.95(3.24) | 85.3(5.02) |
| DELTA | Wilcox | BONF | 0(0) | 1.5(1.19) | 90.7(3.73) | 89.75(4.45) | 0(0) | 0.95(1) | 84.4(5.64) | 83.7(4.75) |
| DELTA | Wilcox | BH | 0(0) | 17.3(4.92) | 90.9(3.61) | 89.75(4.45) | 0(0) | 15.1(3.6) | 84.8(5.63) | 83.95(4.8) |
| DELTA | Nstat | BONF | 0(0) | 3.2(1.54) | 90.85(3.6) | 89.75(4.45) | 0(0) | 2.25(1.48) | 84.5(5.68) | 83.7(4.75) |
| DELTA | Nstat | BH | 0(0) | 19.45(5.34) | 90.9(3.61) | 89.75(4.45) | 0(0) | 17.25(3.27) | 84.75(5.71) | 83.85(4.77) |
| SVA | t | BONF | 0.45(1) | 21.2(14.12) | 80(28.78) | 83(24.52) | 0.35(0.49) | 16.4(17.37) | 93.65(20.24) | 80.5(36.34) |
| SVA | t | BH | 3.55(12.26) | 62.15(11.77) | 82(25.87) | 88.35(18.58) | 4.85(14) | 58.1(24.07) | 94(20.1) | 80.75(36.07) |
| SVA | LIMMA | BONF | 7.75(12.05) | 44.6(11.57) | 80.75(27.53) | 83(24.52) | 10.2(15.51) | 37.7(21.58) | 93.9(20.13) | 80.5(36.34) |
| SVA | LIMMA | BH | 21.25(21.64) | 74.95(14.71) | 82(25.87) | 88.7(18.46) | 30.8(23.71) | 72.25(26.92) | 94(20.1) | 80.75(36.07) |

Table S4: The impact of different sample sizes $n$ on gene selection strategies when the effect size $e$ is fixed and relatively small. Mean (STD) of **false positives** computed from **SIMU2** with 20 repetitions are reported. Effect size: $e = 0.2$. Total number of genes: 1000. Number of differentially expressed genes: 100. Number of permutations for Nstat: 10000. The significance threshold: 0.05.

| | | | **UP 60, DOWN 40** | | | | **UP 90, DOWN 10** | | | |
|---|---|---|---|---|---|---|---|---|---|---|
| Norm | Test | MTP | n=5 | n=10 | n=70 | n=100 | n=5 | n=10 | n=70 | n=100 |
| NONE | t | BONF | 0(0) | 0(0) | 0(0) | 0(0) | 0(0) | 0(0) | 0(0) | 0(0) |
| NONE | t | BH | 0(0) | 0(0) | 0.05(0.22) | 0.05(0.22) | 0(0) | 0(0) | 0.15(0.67) | 0.2(0.89) |
| NONE | LIMMA | BONF | 0(0) | 0(0) | 0(0) | 0.05(0.22) | 0(0) | 0(0) | 0(0) | 0(0) |
| NONE | LIMMA | BH | 0(0) | 0(0) | 0(0) | 44.95(201.02) | 0(0) | 0(0) | 45.45(200.22) | 28.05(125.44) |
| NONE | Wilcox | BONF | 0(0) | 0(0) | 0(0) | 0(0) | 0(0) | 0(0) | 0(0) | 0(0) |
| NONE | Wilcox | BH | 0(0) | 0(0) | 0.1(0.45) | 0.05(0.22) | 0(0) | 0(0) | 0.75(3.35) | 0.15(0.67) |
| NONE | Nstat | BONF | 0(0) | 0(0) | 0(0) | 0(0) | 0(0) | 0(0) | 0(0) | 0(0) |
| NONE | Nstat | BH | 0(0) | 0(0) | 0.1(0.45) | 0.1(0.31) | 0(0) | 0(0) | 0.35(1.57) | 0.1(0.31) |
| GLOBAL | t | BONF | 0(0) | 0.05(0.22) | 0.05(0.22) | 0(0) | 0.05(0.22) | 0.05(0.22) | 0.3(0.47) | 1(0.92) |
| GLOBAL | t | BH | 0.35(0.67) | 3.5(1.57) | 5.35(2.35) | 6.6(2.44) | 0.3(0.73) | 4.35(1.84) | 25.45(3.75) | 44.45(6.19) |
| GLOBAL | LIMMA | BONF | 0.1(0.31) | 0(0) | 0.05(0.22) | 0(0) | 0(0) | 0.1(0.31) | 0.5(0.89) | 1.2(0.77) |
| GLOBAL | LIMMA | BH | 2.05(2.19) | 3.5(2.37) | 6.2(2.69) | 5.8(2.28) | 2.15(1.63) | 6.25(2.31) | 27.95(3.99) | 43.95(7.9) |
| GLOBAL | Wilcox | BONF | 0(0) | 0(0) | 0.05(0.22) | 0.05(0.22) | 0(0) | 0(0) | 0.5(0.61) | 0.7(0.8) |
| GLOBAL | Wilcox | BH | 0(0) | 2.95(1.61) | 6.05(2.46) | 6.25(2.43) | 0(0) | 3.6(1.79) | 24.15(3.3) | 40.85(6.33) |
| GLOBAL | Nstat | BONF | 0(0) | 0.15(0.37) | 0.05(0.22) | 0.05(0.22) | 0(0) | 0.25(0.44) | 0.7(0.73) | 1.25(1.02) |
| GLOBAL | Nstat | BH | 0(0) | 4.05(2.19) | 6.05(2.39) | 6.85(2.54) | 0(0) | 4.6(1.76) | 22.6(2.46) | 39.85(6.43) |
| QUANT | t | BONF | 0(0) | 0.05(0.22) | 0.05(0.22) | 0(0) | 0.05(0.22) | 0.05(0.22) | 0.25(0.44) | 0.65(0.81) |
| QUANT | t | BH | 0.25(0.64) | 2.95(1.47) | 5.4(1.88) | 6.55(2.33) | 0.3(0.73) | 3.45(1.7) | 18.85(3.17) | 32.8(4.49) |
| QUANT | LIMMA | BONF | 0.05(0.22) | 0(0) | 0.05(0.22) | 0(0) | 0(0) | 0.1(0.31) | 0.4(0.75) | 0.9(0.79) |
| QUANT | LIMMA | BH | 1.35(1.66) | 3.3(2.47) | 5.85(2.37) | 5.35(1.76) | 1.25(1.41) | 5.35(2.3) | 22.9(4.63) | 34.2(6.49) |
| QUANT | Wilcox | BONF | 0(0) | 0(0) | 0.05(0.22) | 0.05(0.22) | 0(0) | 0(0) | 0.2(0.41) | 0.25(0.72) |
| QUANT | Wilcox | BH | 0(0) | 2.85(1.57) | 5.55(2.26) | 5.6(2.62) | 0(0) | 2.85(1.39) | 15(2.71) | 23.15(4.46) |
| QUANT | Nstat | BONF | 0(0) | 0.1(0.31) | 0.1(0.31) | 0.05(0.22) | 0(0) | 0.15(0.37) | 0.4(0.6) | 0.85(0.81) |
| QUANT | Nstat | BH | 0(0) | 3.6(1.82) | 6.85(2.72) | 7.5(2.8) | 0(0) | 3.5(1.91) | 16.6(2.91) | 27.65(4.66) |
| RANK | t | BONF | 0.2(0.41) | 0.1(0.31) | 0.05(0.22) | 0.05(0.22) | 0.2(0.52) | 0.15(0.37) | 0.25(0.44) | 0.25(0.72) |
| RANK | t | BH | 1.2(1.24) | 3.85(2.13) | 5.65(2.3) | 5.85(2.28) | 1(1.21) | 4.35(1.9) | 15.45(2.56) | 25.35(4.52) |
| RANK | LIMMA | BONF | 0.05(0.22) | 0(0) | 0.05(0.22) | 0.05(0.22) | 0(0) | 0.15(0.37) | 0.2(0.41) | 0.65(0.49) |
| RANK | LIMMA | BH | 0.25(0.44) | 1.85(1.5) | 6.05(2.39) | 5.3(1.89) | 0(0) | 3.6(1.88) | 18.6(4.16) | 25.85(6.38) |
| RANK | Wilcox | BONF | 0(0) | 0(0) | 0.05(0.22) | 0.05(0.22) | 0(0) | 0(0) | 0.2(0.41) | 0.25(0.72) |
| RANK | Wilcox | BH | 0(0) | 2.85(1.57) | 5.55(2.26) | 5.6(2.62) | 0(0) | 2.85(1.39) | 15(2.71) | 23.15(4.46) |
| RANK | Nstat | BONF | 0(0) | 0.1(0.31) | 0.2(0.41) | 0.05(0.22) | 0(0) | 0.15(0.37) | 0.35(0.59) | 0.55(0.89) |
| RANK | Nstat | BH | 0(0) | 3.25(1.71) | 6.35(2.6) | 6.35(2.54) | 0(0) | 3.4(1.82) | 14.5(3.03) | 23(3.51) |
| DELTA | t | BONF | 0(0) | 0.15(0.37) | 9.9(2.31) | 8.55(2.28) | 0(0) | 0.15(0.37) | 9.9(2.31) | 8.55(2.28) |
| DELTA | t | BH | 0(0) | 2(1.34) | 12.7(3.15) | 10.3(2.99) | 0.05(0.22) | 2.35(1.53) | 12.7(3.15) | 10.25(2.97) |
| DELTA | LIMMA | BONF | 0.1(0.31) | 0.25(0.44) | 8.7(2.96) | 10.35(2.96) | 0.1(0.31) | 0.6(0.82) | 9.25(2.59) | 8.25(2.53) |
| DELTA | LIMMA | BH | 0.6(0.82) | 3.1(1.83) | 11.2(3.33) | 12.3(2.64) | 0.75(0.72) | 4.1(2.31) | 11.5(2.84) | 9.7(3.23) |
| DELTA | Wilcox | BONF | 0(0) | 0.1(0.31) | 9.9(2.31) | 8.55(2.28) | 0(0) | 0(0) | 9.9(2.31) | 8.55(2.28) |
| DELTA | Wilcox | BH | 0(0) | 1.6(1.19) | 12.6(2.74) | 10.85(3.07) | 0(0) | 1.95(1.43) | 12.6(2.74) | 10.75(3.08) |
| DELTA | Nstat | BONF | 0(0) | 0.25(0.44) | 10(2.41) | 8.55(2.28) | 0(0) | 0.15(0.37) | 10(2.41) | 8.55(2.28) |
| DELTA | Nstat | BH | 0(0) | 2(1.59) | 12.45(2.54) | 10.85(3.36) | 0(0) | 2.25(1.41) | 12.4(2.64) | 10.8(3.25) |
| SVA | t | BONF | 0.25(0.72) | 10.8(23.75) | 0.15(0.37) | 0.1(0.31) | 0.25(0.72) | 22.4(49.11) | 0(0) | 0.7(1.72) |
| SVA | t | BH | 10.95(48.27) | 159.8(300.59) | 4.55(6.84) | 53.55(200.07) | 3.15(12.1) | 172.1(341.4) | 49.25(199.57) | 40.3(109.31) |
| SVA | LIMMA | BONF | 1.1(4.69) | 40(91.46) | 0.05(0.22) | 0.15(0.37) | 0.7(1.95) | 66.1(142.48) | 0(0) | 0.5(1.15) |
| SVA | LIMMA | BH | 26.05(110.87) | 177.7(317.81) | 4.95(7.16) | 53.25(200.04) | 23.75(68.99) | 180.7(350.07) | 49.25(199.34) | 45.3(126.5) |

Table S5: The impact of different sample sizes $n$ on gene selection strategies when the effect size $e$ is fixed and relatively large. Mean (STD) of **true positives** computed from **SIMU2** with 20 repetitions are reported. Effect size: $e = 1.8$. Total number of genes: 1000. Number of differentially expressed genes: 100. Number of permutations for Nstat: 10000. The significance threshold: 0.05.

| | | | **UP 60, DOWN 40** | | | | **UP 90, DOWN 10** | | | |
|---|---|---|---|---|---|---|---|---|---|---|
| Norm | Test | MTP | n=5 | n=10 | n=70 | n=100 | n=5 | n=10 | n=70 | n=100 |
| NONE | t | BONF | 68.9(29.32) | 100(0) | 100(0) | 100(0) | 53.6(39.02) | 100(0) | 100(0) | 100(0) |
| NONE | t | BH | 100(0) | 100(0) | 100(0) | 100(0) | 100(0) | 100(0) | 100(0) | 100(0) |
| NONE | LIMMA | BONF | 100(0) | 100(0) | 100(0) | 100(0) | 100(0) | 100(0) | 100(0) | 100(0) |
| NONE | LIMMA | BH | 100(0) | 100(0) | 100(0) | 100(0) | 100(0) | 100(0) | 100(0) | 100(0) |
| NONE | Wilcox | BONF | 0(0) | 99.9(0.45) | 100(0) | 100(0) | 0(0) | 99.85(0.67) | 100(0) | 100(0) |
| NONE | Wilcox | BH | 5(22.36) | 100(0) | 100(0) | 100(0) | 0(0) | 100(0) | 100(0) | 100(0) |
| NONE | Nstat | BONF | 0.05(0.22) | 99.65(1.57) | 100(0) | 100(0) | 0(0) | 99.45(2.46) | 100(0) | 100(0) |
| NONE | Nstat | BH | 5.05(22.35) | 100(0) | 100(0) | 100(0) | 0(0) | 100(0) | 100(0) | 100(0) |
| GLOBAL | t | BONF | 100(0) | 100(0) | 100(0) | 100(0) | 100(0) | 100(0) | 100(0) | 100(0) |
| GLOBAL | t | BH | 100(0) | 100(0) | 100(0) | 100(0) | 100(0) | 100(0) | 100(0) | 100(0) |
| GLOBAL | LIMMA | BONF | 100(0) | 100(0) | 100(0) | 100(0) | 100(0) | 100(0) | 100(0) | 100(0) |
| GLOBAL | LIMMA | BH | 100(0) | 100(0) | 100(0) | 100(0) | 100(0) | 100(0) | 100(0) | 100(0) |
| GLOBAL | Wilcox | BONF | 0(0) | 100(0) | 100(0) | 100(0) | 0(0) | 100(0) | 100(0) | 100(0) |
| GLOBAL | Wilcox | BH | 0(0) | 100(0) | 100(0) | 100(0) | 100(0) | 100(0) | 100(0) | 100(0) |
| GLOBAL | Nstat | BONF | 0(0) | 100(0) | 100(0) | 100(0) | 0(0) | 100(0) | 100(0) | 100(0) |
| GLOBAL | Nstat | BH | 0(0) | 100(0) | 100(0) | 100(0) | 100(0) | 100(0) | 100(0) | 100(0) |
| QUANT | t | BONF | 59(4.75) | 95.05(2.67) | 100(0) | 100(0) | 59(4.41) | 92.25(2) | 100(0) | 100(0) |
| QUANT | t | BH | 83.75(2.49) | 99.95(0.22) | 100(0) | 100(0) | 76.15(5.11) | 99.9(0.31) | 100(0) | 100(0) |
| QUANT | LIMMA | BONF | 64.15(4.76) | 98.05(1.15) | 100(0) | 100(0) | 61.55(4.33) | 95.6(2.01) | 100(0) | 100(0) |
| QUANT | LIMMA | BH | 94.1(2.61) | 100(0) | 100(0) | 100(0) | 91.6(2.66) | 99.9(0.31) | 100(0) | 100(0) |
| QUANT | Wilcox | BONF | 0(0) | 67.7(4.52) | 100(0) | 100(0) | 0(0) | 56.25(5.04) | 100(0) | 100(0) |
| QUANT | Wilcox | BH | 0(0) | 97.45(1.82) | 100(0) | 100(0) | 0(0) | 93(2.34) | 100(0) | 100(0) |
| QUANT | Nstat | BONF | 0(0) | 82.8(3.3) | 100(0) | 100(0) | 0(0) | 72.55(3.71) | 100(0) | 100(0) |
| QUANT | Nstat | BH | 0(0) | 99.9(0.45) | 100(0) | 100(0) | 0(0) | 99.45(0.6) | 100(0) | 100(0) |
| RANK | t | BONF | 5.85(1.79) | 46.1(3.97) | 100(0) | 100(0) | 5.65(2.06) | 40.65(4.42) | 100(0) | 100(0) |
| RANK | t | BH | 23.6(5.08) | 97.85(1.42) | 100(0) | 100(0) | 23.75(7.29) | 96.4(1.54) | 100(0) | 100(0) |
| RANK | LIMMA | BONF | 4.8(1.96) | 42.3(4.74) | 100(0) | 100(0) | 3.35(2.08) | 36.7(4.26) | 100(0) | 100(0) |
| RANK | LIMMA | BH | 27.45(8.62) | 91.65(2.74) | 100(0) | 100(0) | 22.6(8.42) | 89.6(2.28) | 100(0) | 100(0) |
| RANK | Wilcox | BONF | 0(0) | 67.7(4.52) | 100(0) | 100(0) | 0(0) | 56.25(5.04) | 100(0) | 100(0) |
| RANK | Wilcox | BH | 0(0) | 97.45(1.82) | 100(0) | 100(0) | 0(0) | 93(2.34) | 100(0) | 100(0) |
| RANK | Nstat | BONF | 0(0) | 82.25(3.54) | 100(0) | 100(0) | 0(0) | 71.7(3.51) | 100(0) | 100(0) |
| RANK | Nstat | BH | 0(0) | 99.85(0.49) | 100(0) | 100(0) | 0(0) | 99.3(0.66) | 100(0) | 100(0) |
| DELTA | t | BONF | 90(2.88) | 91.55(3.83) | 89.4(3.41) | 89.75(4.33) | 85.5(6.06) | 86.45(3.97) | 83.7(4.43) | 84.1(5.17) |
| DELTA | t | BH | 90.1(2.85) | 91.75(3.91) | 89.4(3.41) | 89.95(4.33) | 85.7(5.93) | 86.65(4.09) | 83.8(4.31) | 84.4(4.62) |
| DELTA | LIMMA | BONF | 90(2.88) | 90.65(3.28) | 90.95(3.58) | 88.8(3.58) | 85.5(6.06) | 84.3(3.59) | 85.4(5.86) | 85.2(4.6) |
| DELTA | LIMMA | BH | 90.1(2.85) | 90.65(3.28) | 91.05(3.58) | 89(3.4) | 85.85(6.01) | 84.5(3.47) | 85.5(5.71) | 85.2(4.6) |
| DELTA | Wilcox | BONF | 0(0) | 91.55(3.83) | 89.4(3.41) | 89.75(4.33) | 0(0) | 86.45(3.97) | 83.7(4.43) | 84.1(5.17) |
| DELTA | Wilcox | BH | 90.1(2.85) | 91.75(3.91) | 89.4(3.41) | 89.95(4.33) | 85.6(6.03) | 86.65(4.09) | 83.9(4.39) | 84.3(4.87) |
| DELTA | Nstat | BONF | 0(0) | 91.55(3.83) | 89.4(3.41) | 89.75(4.33) | 0(0) | 86.45(3.97) | 83.7(4.43) | 84.1(5.17) |
| DELTA | Nstat | BH | 90.1(2.85) | 91.75(3.91) | 89.4(3.41) | 89.95(4.33) | 85.6(6.03) | 86.65(4.09) | 83.9(4.39) | 84.4(4.62) |
| SVA | t | BONF | 93.85(14.89) | 100(0) | 100(0) | 100(0) | 97.75(8.37) | 100(0) | 100(0) | 100(0) |
| SVA | t | BH | 100(0) | 100(0) | 100(0) | 100(0) | 100(0) | 100(0) | 100(0) | 100(0) |
| SVA | LIMMA | BONF | 100(0) | 100(0) | 100(0) | 100(0) | 100(0) | 100(0) | 100(0) | 100(0) |
| SVA | LIMMA | BH | 100(0) | 100(0) | 100(0) | 100(0) | 100(0) | 100(0) | 100(0) | 100(0) |

Table S6: The impact of different sample sizes $n$ on gene selection strategies when the effect size $e$ is fixed and relatively large. Mean (STD) of **false positives** computed from **SIMU2** with 20 repetitions are reported. Effect size: $e = 1.8$. Total number of genes: 1000. Number of differentially expressed genes: 100. Number of permutations for Nstat: 10000. The significance threshold: 0.05.

| | | | **UP 60, DOWN 40** | | | | **UP 90, DOWN 10** | | | |
|---|---|---|---|---|---|---|---|---|---|---|
| Norm | Test | MTP | n=5 | n=10 | n=70 | n=100 | n=5 | n=10 | n=70 | n=100 |
| NONE | t | BONF | 0(0) | 0(0) | 0(0) | 0(0) | 0(0) | 0(0) | 0(0) | 0(0) |
| NONE | t | BH | 44.65(198.74) | 45.2(199.33) | 0.4(1.79) | 43.75(195.66) | 0.3(1.34) | 45.2(199.33) | 0.4(1.79) | 43.75(195.66) |
| NONE | LIMMA | BONF | 5.3(23.7) | 0(0) | 0(0) | 0(0) | 0(0) | 1.35(6.04) | 0(0) | 0(0) |
| NONE | LIMMA | BH | 45.25(201.19) | 0(0) | 87.15(268.09) | 0(0) | 1(4.47) | 86.45(266.17) | 44.35(195.07) | 0.1(0.45) |
| NONE | Wilcox | BONF | 0(0) | 0.1(0.45) | 0(0) | 0.05(0.22) | 0(0) | 0.1(0.45) | 0(0) | 0.05(0.22) |
| NONE | Wilcox | BH | 43.15(192.97) | 43.05(187.13) | 0.05(0.22) | 44.6(199.46) | 0(0) | 43.05(187.13) | 0.05(0.22) | 44.6(199.46) |
| NONE | Nstat | BONF | 0(0) | 0.2(0.89) | 0(0) | 0.2(0.89) | 0(0) | 0.2(0.89) | 0(0) | 0.2(0.89) |
| NONE | Nstat | BH | 44(196.77) | 43.1(190.63) | 0.3(0.92) | 44.35(198.34) | 0(0) | 43.1(190.63) | 0.3(0.92) | 44.35(198.34) |
| GLOBAL | t | BONF | 0.05(0.22) | 0(0) | 12.45(3.98) | 31.6(4.33) | 2.1(1.17) | 36.3(6.2) | 899.65(0.67) | 900(0) |
| GLOBAL | t | BH | 8.85(3.3) | 16.2(3.85) | 302.95(11.84) | 488.95(12.23) | 212.85(17.06) | 672.3(12.83) | 900(0) | 900(0) |
| GLOBAL | LIMMA | BONF | 0.05(0.22) | 0.45(0.6) | 15.55(4.32) | 34.4(6.32) | 19.35(3.72) | 106.4(9.69) | 899.75(0.44) | 900(0) |
| GLOBAL | LIMMA | BH | 13.15(3.18) | 19.3(4.34) | 312.75(10.48) | 500.05(12.51) | 374.5(17.97) | 725.75(10.08) | 900(0) | 900(0) |
| GLOBAL | Wilcox | BONF | 0(0) | 0.05(0.22) | 11.25(3.67) | 28.65(4.73) | 0(0) | 25.05(3.63) | 899.6(0.6) | 900(0) |
| GLOBAL | Wilcox | BH | 0(0) | 14.6(3.49) | 280.15(12.07) | 465.85(15.86) | 131.65(30.79) | 641.5(10.97) | 900(0) | 900(0) |
| GLOBAL | Nstat | BONF | 0(0) | 0.1(0.31) | 14.55(3.52) | 34.4(4.36) | 0.05(0.22) | 48(5.85) | 898.9(0.97) | 900(0) |
| GLOBAL | Nstat | BH | 0(0) | 16.5(3.72) | 273.2(12.11) | 455.85(16.46) | 133.3(9.56) | 645.7(15.31) | 900(0) | 900(0) |
| QUANT | t | BONF | 0(0) | 0(0) | 0(0) | 0.05(0.22) | 0.1(0.31) | 0(0) | 1.45(1.15) | 10.75(3.61) |
| QUANT | t | BH | 2.15(1.66) | 1.65(1.27) | 6.75(2.9) | 9.7(3.29) | 1.95(1.05) | 2.15(1.27) | 300.9(18.71) | 541(14.14) |
| QUANT | LIMMA | BONF | 0(0) | 0(0) | 0(0) | 0.1(0.31) | 0(0) | 0(0) | 2.7(1.17) | 15.25(3.95) |
| QUANT | LIMMA | BH | 0.75(0.55) | 0.7(0.73) | 7.05(3.02) | 10.3(2.85) | 0.65(0.81) | 0.55(0.76) | 316.05(9.38) | 546.3(12.77) |
| QUANT | Wilcox | BONF | 0(0) | 0(0) | 0(0) | 0.2(0.52) | 0(0) | 0(0) | 0.5(0.83) | 1.1(1.17) |
| QUANT | Wilcox | BH | 0(0) | 4.45(2.5) | 5.5(2.74) | 5.8(2.59) | 0(0) | 4.85(1.76) | 24.35(4.89) | 37.85(6.87) |
| QUANT | Nstat | BONF | 0(0) | 0.05(0.22) | 1.6(1.47) | 10(3.23) | 0(0) | 0(0) | 15(3.28) | 60.4(4.82) |
| QUANT | Nstat | BH | 0(0) | 5.2(1.94) | 252.35(20.4) | 591.45(14.9) | 0(0) | 6.35(1.63) | 537.35(12.68) | 768.05(11.28) |
| RANK | t | BONF | 0.05(0.22) | 0.05(0.22) | 0.05(0.22) | 0.25(0.55) | 0.35(0.75) | 0.05(0.22) | 0.75(1.25) | 1.5(1.43) |
| RANK | t | BH | 2(1.45) | 5.35(2.08) | 5.35(2.89) | 6.4(3.17) | 3.7(2.34) | 7.85(3.01) | 27.55(5.07) | 41.45(6.44) |
| RANK | LIMMA | BONF | 0(0) | 0(0) | 0.1(0.31) | 0.1(0.31) | 0.1(0.31) | 0.05(0.22) | 0.9(0.79) | 1.4(0.94) |
| RANK | LIMMA | BH | 1(0.86) | 4.8(2.75) | 6.5(2.04) | 7.55(2.37) | 1.7(1.38) | 6.35(2.56) | 31.9(6.16) | 48.6(9.59) |
| RANK | Wilcox | BONF | 0(0) | 0(0) | 0(0) | 0.2(0.52) | 0(0) | 0(0) | 0.5(0.83) | 1.1(1.17) |
| RANK | Wilcox | BH | 0(0) | 4.45(2.5) | 5.5(2.74) | 5.8(2.59) | 0(0) | 4.85(1.76) | 24.35(4.89) | 37.85(6.87) |
| RANK | Nstat | BONF | 0(0) | 0.05(0.22) | 0.05(0.22) | 0.3(0.57) | 0(0) | 0(0) | 0.95(1.28) | 1.5(1.4) |
| RANK | Nstat | BH | 0(0) | 4.8(1.58) | 6.65(3.42) | 7.65(3.27) | 0(0) | 5.9(2.34) | 23.1(5.27) | 34.7(6.8) |
| DELTA | t | BONF | 9.3(2.68) | 9.35(2.64) | 8.95(2.89) | 8.55(3.22) | 8.7(3.36) | 9.35(2.64) | 8.95(2.89) | 8.55(3.22) |
| DELTA | t | BH | 12.55(2.7) | 11.9(2.57) | 11.05(3.53) | 11.1(4.25) | 12.5(4.3) | 11.9(2.57) | 10.95(3.46) | 11.05(4.29) |
| DELTA | LIMMA | BONF | 9.25(2.59) | 9.85(2.25) | 8.3(2.08) | 7.75(1.97) | 8.7(3.36) | 9.75(2.92) | 9.05(3.33) | 8.55(1.76) |
| DELTA | LIMMA | BH | 12.7(3.11) | 12.3(2.77) | 10.75(2.92) | 10.05(2.56) | 13.05(4.62) | 11.85(3.05) | 11.55(3.49) | 11.65(2.81) |
| DELTA | Wilcox | BONF | 0(0) | 9.35(2.64) | 8.95(2.89) | 8.5(3.14) | 0(0) | 9.35(2.64) | 8.95(2.89) | 8.5(3.14) |
| DELTA | Wilcox | BH | 11.6(2.5) | 11.9(2.95) | 11(3.74) | 10.7(4) | 11.65(3.99) | 11.85(3.01) | 11(3.74) | 10.45(3.94) |
| DELTA | Nstat | BONF | 0(0) | 9.35(2.64) | 8.95(2.89) | 8.5(3.14) | 0(0) | 9.35(2.64) | 8.95(2.89) | 8.5(3.14) |
| DELTA | Nstat | BH | 11.75(2.45) | 12.25(3.09) | 10.95(3.3) | 10.55(3.75) | 11.65(3.99) | 12.2(3.04) | 10.95(3.3) | 10.55(3.75) |
| SVA | t | BONF | 0.05(0.22) | 0.05(0.22) | 0.4(0.99) | 0(0) | 0.1(0.45) | 0.1(0.45) | 0.7(1.95) | 0.05(0.22) |
| SVA | t | BH | 52.6(197.29) | 10.35(13.82) | 105.7(262.43) | 4.65(3.39) | 5.45(3.82) | 86.3(255.33) | 65.6(194.79) | 4.8(4.29) |
| SVA | LIMMA | BONF | 5.7(23.64) | 0.1(0.31) | 0.45(1.1) | 0(0) | 0.15(0.37) | 1.45(6.03) | 0.7(2.08) | 0(0) |
| SVA | LIMMA | BH | 62.9(199.8) | 13.1(18.57) | 107.6(264.3) | 4.4(3.07) | 5.9(6.25) | 90.5(264.84) | 66.55(196.06) | 4.55(4.99) |

Table S7: The impact of different sample sizes $n$ on gene selection strategies with simulation based on biological data. Mean (STD) of **true positives** computed from **SIMU-BIO** with 20 repetitions are reported. Total number of genes: 9005. Number of permutations for Nstat: 100000. The significance threshold: 0.05.

| | | | **Unbalanced (677:57)** | | | | **Balanced (259:287)** | | | |
|---|---|---|---|---|---|---|---|---|---|---|
| Norm | Test | MTP | n=5 | n=10 | n=40 | n=79 | n=5 | n=10 | n=30 | n=45 |
| NONE | t | BONF | 0.4(1.39) | 0.4(1.57) | 16.15(15.14) | 119.65(78.78) | 0(0) | 2.65(5.97) | 43(35.57) | 98.55(35.87) |
| NONE | t | BH | 33.9(147.88) | 35(152.11) | 80.1(87.52) | 348.45(209.41) | 8.8(39.35) | 38.55(92.4) | 136.9(90.42) | 267.75(61.59) |
| NONE | LIMMA | BONF | 32.2(111.54) | 42.2(112.49) | 74.4(34.05) | 165.25(91.35) | 18.6(13.82) | 52.15(43.14) | 117.75(48.56) | 154.2(36.98) |
| NONE | LIMMA | BH | 63.4(161.77) | 78.25(149) | 174.1(112.75) | 381.5(197.64) | 45.65(58.1) | 102.9(98.29) | 227.6(79.36) | 318.75(56.76) |
| NONE | Wilcox | BONF | 0(0) | 0(0) | 15(13.83) | 121.2(76.72) | 0(0) | 0(0) | 38.85(34.31) | 97.6(37.87) |
| NONE | Wilcox | BH | 32(143.11) | 34.15(150.85) | 71.7(64.33) | 356.7(217.61) | 0(0) | 34.3(89.26) | 128.25(85.9) | 258.05(64.32) |
| NONE | Nstat | BONF | 0(0) | 0.3(0.73) | 15.75(14.19) | 131.55(90.41) | 0(0) | 0.65(1.39) | 44.15(32.92) | 98.85(35.52) |
| NONE | Nstat | BH | 32.7(146.24) | 34.6(151.23) | 79.15(70.57) | 356.95(221) | 0(0) | 34.4(90.95) | 128.1(85.5) | 256.25(57.36) |
| GLOBAL | t | BONF | 0.25(0.55) | 5.8(15.66) | 238.8(113) | 529.95(84.91) | 0.4(0.75) | 18.9(45.27) | 234.95(113.15) | 372.35(55.92) |
| GLOBAL | t | BH | 28.45(87.94) | 76.25(118.13) | 546.2(122.25) | 698.15(34.02) | 0.8(2.21) | 100.25(146.59) | 437.1(76.57) | 490.25(34.07) |
| GLOBAL | LIMMA | BONF | 3.05(7.84) | 10.7(25.05) | 254.2(117.42) | 535.95(84.49) | 1.1(1.48) | 30.75(68.13) | 250.05(112.82) | 378.35(54.21) |
| GLOBAL | LIMMA | BH | 53.3(143.64) | 120.55(149.75) | 556.3(117.9) | 699.4(33.04) | 37.6(57.78) | 151.05(148.92) | 443.9(71.63) | 492.35(33.5) |
| GLOBAL | Wilcox | BONF | 0(0) | 0(0) | 289.3(121.91) | 568.65(68.78) | 0(0) | 0(0) | 256.5(124.46) | 406.9(49.46) |
| GLOBAL | Wilcox | BH | 0(0) | 75.85(124.06) | 578.35(104.12) | 705.75(24.45) | 0(0) | 97.5(149.74) | 460.6(61.8) | 503.5(23.79) |
| GLOBAL | Nstat | BONF | 0.1(0.31) | 2.95(6.01) | 294.3(119.48) | 565.2(67.6) | 0(0) | 4.7(7.41) | 277.8(106.54) | 405.95(44.55) |
| GLOBAL | Nstat | BH | 0.1(0.31) | 87.4(131.84) | 578.4(105.48) | 704.6(24.22) | 0(0) | 116.3(151.71) | 464.3(51.85) | 501.5(26.12) |
| QUANT | t | BONF | 0.35(0.81) | 11.45(26.89) | 312.75(63.38) | 524.65(15.57) | 0.3(0.8) | 31.4(72.82) | 319.8(122.08) | 463.65(40.16) |
| QUANT | t | BH | 14.85(48.71) | 129.7(136.66) | 554.1(36.43) | 638.8(8.52) | 0.7(2.3) | 154.15(169.4) | 503.95(35.53) | 532.05(8.04) |
| QUANT | LIMMA | BONF | 3.35(9.51) | 23.75(40.79) | 325.85(63) | 528.2(15.52) | 2.1(3.09) | 47.2(101.05) | 336.3(117.13) | 468.6(38.04) |
| QUANT | LIMMA | BH | 45.05(107.86) | 180.5(154.45) | 559(34.93) | 640.15(8.29) | 53.75(85.4) | 215.95(171.15) | 507.4(32.68) | 532.7(7.57) |
| QUANT | Wilcox | BONF | 0(0) | 0(0) | 332.2(66.38) | 543.45(14.16) | 0(0) | 0(0) | 314.55(133.69) | 471.05(41.31) |
| QUANT | Wilcox | BH | 0(0) | 113.65(130.05) | 567.95(34.94) | 642.9(7.49) | 0(0) | 136.2(168.22) | 509.8(34.03) | 534.85(7.18) |
| QUANT | Nstat | BONF | 0(0) | 1.75(2.02) | 349.9(64.49) | 550.1(13.46) | 0(0) | 4.4(8.39) | 338.1(119.96) | 480.15(35.2) |
| QUANT | Nstat | BH | 0(0) | 129(137.67) | 575.5(31.45) | 647.1(7.63) | 0(0) | 162.45(167.93) | 514.95(25.92) | 535.85(6.45) |
| RANK | t | BONF | 1(2.71) | 10.5(21.14) | 306.35(57.93) | 521.1(12.32) | 2(3.6) | 30.4(62.3) | 300.3(111.2) | 448.15(36.96) |
| RANK | t | BH | 11.35(33.9) | 95.15(117.31) | 559.05(31.96) | 641.8(9.13) | 8.35(16.16) | 132.45(153.5) | 502.15(30.03) | 529.75(7.79) |
| RANK | LIMMA | BONF | 2.6(6.92) | 12.6(22.51) | 279.6(50.49) | 475.75(13.04) | 3.75(5.74) | 36.1(67.48) | 281.8(98.36) | 409.5(31.39) |
| RANK | LIMMA | BH | 13.4(36.73) | 80.65(94.36) | 485.9(30.05) | 576.2(7.68) | 20.45(33.5) | 142.15(131.73) | 446.9(27.84) | 483.45(9.65) |
| RANK | Wilcox | BONF | 0(0) | 0(0) | 332.2(66.38) | 543.45(14.16) | 0(0) | 0(0) | 314.55(133.69) | 471.05(41.31) |
| RANK | Wilcox | BH | 0(0) | 113.65(130.05) | 567.95(34.94) | 642.9(7.49) | 0(0) | 136.2(168.22) | 509.8(34.03) | 534.85(7.18) |
| RANK | Nstat | BONF | 0(0) | 1.05(1.88) | 374.3(60.28) | 563.05(11.54) | 0(0) | 1.65(2.87) | 355.95(110.37) | 488.45(32.28) |
| RANK | Nstat | BH | 0(0) | 136.2(141.29) | 586.1(27.28) | 653.35(7.26) | 0(0) | 157.25(168.67) | 519.2(22.05) | 537.4(5.16) |
| DELTA | t | BONF | 0.05(0.22) | 2.05(6.66) | 110.6(69.86) | 291.15(56.22) | 0.2(0.41) | 10.3(24.4) | 113.7(80.34) | 203.25(72.17) |
| DELTA | t | BH | 8.45(27.1) | 35(55.97) | 328.55(95.91) | 460.1(33.65) | 3.5(5.81) | 75(113.88) | 291.2(88.02) | 354.55(48.63) |
| DELTA | LIMMA | BONF | 0.7(2.15) | 3.75(9.72) | 117.8(72.85) | 295.7(55.94) | 0.9(1.29) | 16.6(36.4) | 121.8(82.95) | 209.55(71.66) |
| DELTA | LIMMA | BH | 22.35(60.21) | 55.35(74.75) | 336.25(94.71) | 461.95(33.54) | 29.55(39.71) | 97.4(119.84) | 298.05(86.34) | 358.4(46.96) |
| DELTA | Wilcox | BONF | 0(0) | 1.3(4) | 132.9(78.81) | 325.45(48.78) | 0(0) | 2.85(5) | 123.8(86.44) | 226.9(73.66) |
| DELTA | Wilcox | BH | 0(0) | 33.1(53.52) | 357(89.6) | 477(29.97) | 0(0) | 71.15(110.83) | 306.95(87.83) | 375.45(39.73) |
| DELTA | Nstat | BONF | 0(0) | 2.1(4.9) | 143.85(80.74) | 329.2(47.55) | 0(0) | 6(11.15) | 134.65(83.64) | 234.4(70.87) |
| DELTA | Nstat | BH | 0(0) | 37.85(60.65) | 355.5(90.74) | 475(29.73) | 0(0) | 80.8(115.99) | 311(81.16) | 374.8(39.69) |
| SVA | t | BONF | 1.25(4.45) | 13.8(28.86) | 396.7(189.31) | 642.2(127.84) | 0.35(0.93) | 22.75(48.88) | 292.15(132.89) | 418.7(87.49) |
| SVA | t | BH | 84.15(202) | 275.85(220.65) | 623.55(139.32) | 706.8(71.81) | 57.4(108.28) | 227.3(185.91) | 479.85(64.29) | 502.5(57.35) |
| SVA | LIMMA | BONF | 115.3(204.37) | 61.15(132.43) | 414.7(187.5) | 644.4(128.03) | 7.35(12.49) | 81.65(103.71) | 312.15(127.91) | 424.75(86.04) |
| SVA | LIMMA | BH | 235.25(292.35) | 334.3(235.51) | 625.95(140.33) | 706.95(72.34) | 137.8(160.13) | 269.7(170.25) | 483.4(61.45) | 502.65(58.09) |

Table S8: The impact of different sample sizes $n$ on gene selection strategies with simulation based on biological data. Mean (STD) of **false positives** computed from **SIMU-BIO** with 20 repetitions are reported. Total number of genes: 9005. Number of permutations for Nstat: 100000. The significance threshold: 0.05.

| | | | **Unbalanced (677:57)** | | | | **Balanced (259:287)** | | | |
|---|---|---|---|---|---|---|---|---|---|---|
| Norm | Test | MTP | n=5 | n=10 | n=40 | n=79 | n=5 | n=10 | n=30 | n=45 |
| NONE | t | BONF | 0.4(1.39) | 0.4(1.57) | 16.15(15.14) | 119.65(78.78) | 0(0) | 2.65(5.97) | 43(35.57) | 98.55(35.87) |
| NONE | t | BH | 33.9(147.88) | 35(152.11) | 80.1(87.52) | 348.45(209.41) | 8.8(39.35) | 38.55(92.4) | 136.9(90.42) | 267.75(61.59) |
| NONE | LIMMA | BONF | 32.2(111.54) | 42.2(112.49) | 74.4(34.05) | 165.25(91.35) | 18.6(13.82) | 52.15(43.14) | 117.75(48.56) | 154.2(36.98) |
| NONE | LIMMA | BH | 63.4(161.77) | 78.25(149) | 174.1(112.75) | 381.5(197.64) | 45.65(58.1) | 102.9(98.29) | 227.6(79.36) | 318.75(56.76) |
| NONE | Wilcox | BONF | 0(0) | 0(0) | 15(13.83) | 121.2(76.72) | 0(0) | 0(0) | 38.85(34.31) | 97.6(37.87) |
| NONE | Wilcox | BH | 32(143.11) | 34.15(150.85) | 71.7(64.33) | 356.7(217.61) | 0(0) | 34.3(89.26) | 128.25(85.9) | 258.05(64.32) |
| NONE | Nstat | BONF | 0(0) | 0.3(0.73) | 15.75(14.19) | 131.55(90.41) | 0(0) | 0.65(1.39) | 44.15(32.92) | 98.85(35.52) |
| NONE | Nstat | BH | 32.7(146.24) | 34.6(151.23) | 79.15(70.57) | 356.95(221) | 0(0) | 34.4(90.95) | 128.1(85.5) | 256.25(57.36) |
| GLOBAL | t | BONF | 0.25(0.55) | 5.8(15.66) | 238.8(113) | 529.95(84.91) | 0.4(0.75) | 18.9(45.27) | 234.95(113.15) | 372.35(55.92) |
| GLOBAL | t | BH | 28.45(87.94) | 76.25(118.13) | 546.2(122.25) | 698.15(34.02) | 0.8(2.21) | 100.25(146.59) | 437.1(76.57) | 490.25(34.07) |
| GLOBAL | LIMMA | BONF | 3.05(7.84) | 10.7(25.05) | 254.2(117.42) | 535.95(84.49) | 1.1(1.48) | 30.75(68.13) | 250.05(112.82) | 378.35(54.21) |
| GLOBAL | LIMMA | BH | 53.3(143.64) | 120.55(149.75) | 556.3(117.9) | 699.4(33.04) | 37.6(57.78) | 151.05(148.92) | 443.9(71.63) | 492.35(33.5) |
| GLOBAL | Wilcox | BONF | 0(0) | 0(0) | 289.3(121.91) | 568.65(68.78) | 0(0) | 0(0) | 256.5(124.46) | 406.9(49.46) |
| GLOBAL | Wilcox | BH | 0(0) | 75.85(124.06) | 578.35(104.12) | 705.75(24.45) | 0(0) | 97.5(149.74) | 460.6(61.8) | 503.5(23.79) |
| GLOBAL | Nstat | BONF | 0.1(0.31) | 2.95(6.01) | 294.3(119.48) | 565.2(67.6) | 0(0) | 4.7(7.41) | 277.8(106.54) | 405.95(44.55) |
| GLOBAL | Nstat | BH | 0.1(0.31) | 87.4(131.84) | 578.4(105.48) | 704.6(24.22) | 0(0) | 116.3(151.71) | 464.3(51.85) | 501.5(26.12) |
| QUANT | t | BONF | 0.35(0.81) | 11.45(26.89) | 312.75(63.38) | 524.65(15.57) | 0.3(0.8) | 31.4(72.82) | 319.8(122.08) | 463.65(40.16) |
| QUANT | t | BH | 14.85(48.71) | 129.7(136.66) | 554.1(36.43) | 638.8(8.52) | 0.7(2.3) | 154.15(169.4) | 503.95(35.53) | 532.05(8.04) |
| QUANT | LIMMA | BONF | 3.35(9.51) | 23.75(40.79) | 325.85(63) | 528.2(15.52) | 2.1(3.09) | 47.2(101.05) | 336.3(117.13) | 468.6(38.04) |
| QUANT | LIMMA | BH | 45.05(107.86) | 180.5(154.45) | 559(34.93) | 640.15(8.29) | 53.75(85.4) | 215.95(171.15) | 507.4(32.68) | 532.7(7.57) |
| QUANT | Wilcox | BONF | 0(0) | 0(0) | 332.2(66.38) | 543.45(14.16) | 0(0) | 0(0) | 314.55(133.69) | 471.05(41.31) |
| QUANT | Wilcox | BH | 0(0) | 113.65(130.05) | 567.95(34.94) | 642.9(7.49) | 0(0) | 136.2(168.22) | 509.8(34.03) | 534.85(7.18) |
| QUANT | Nstat | BONF | 0(0) | 1.75(2.02) | 349.9(64.49) | 550.1(13.46) | 0(0) | 4.4(8.39) | 338.1(119.96) | 480.15(35.2) |
| QUANT | Nstat | BH | 0(0) | 129(137.67) | 575.5(31.45) | 647.1(7.63) | 0(0) | 162.45(167.93) | 514.95(25.92) | 535.85(6.45) |
| RANK | t | BONF | 1(2.71) | 10.5(21.14) | 306.35(57.93) | 521.1(12.32) | 2(3.6) | 30.4(62.3) | 300.3(111.2) | 448.15(36.96) |
| RANK | t | BH | 11.35(33.9) | 95.15(117.31) | 559.05(31.96) | 641.8(9.13) | 8.35(16.16) | 132.45(153.5) | 502.15(30.03) | 529.75(7.79) |
| RANK | LIMMA | BONF | 2.6(6.92) | 12.6(22.51) | 279.6(50.49) | 475.75(13.04) | 3.75(5.74) | 36.1(67.48) | 281.8(98.36) | 409.5(31.39) |
| RANK | LIMMA | BH | 13.4(36.73) | 80.65(94.36) | 485.9(30.05) | 576.2(7.68) | 20.45(33.5) | 142.15(131.73) | 446.9(27.84) | 483.45(9.65) |
| RANK | Wilcox | BONF | 0(0) | 0(0) | 332.2(66.38) | 543.45(14.16) | 0(0) | 0(0) | 314.55(133.69) | 471.05(41.31) |
| RANK | Wilcox | BH | 0(0) | 113.65(130.05) | 567.95(34.94) | 642.9(7.49) | 0(0) | 136.2(168.22) | 509.8(34.03) | 534.85(7.18) |
| RANK | Nstat | BONF | 0(0) | 1.05(1.88) | 374.3(60.28) | 563.05(11.54) | 0(0) | 1.65(2.87) | 355.95(110.37) | 488.45(32.28) |
| RANK | Nstat | BH | 0(0) | 136.2(141.29) | 586.1(27.28) | 653.35(7.26) | 0(0) | 157.25(168.67) | 519.2(22.05) | 537.4(5.16) |
| DELTA | t | BONF | 0.05(0.22) | 2.05(6.66) | 110.6(69.86) | 291.15(56.22) | 0.2(0.41) | 10.3(24.4) | 113.7(80.34) | 203.25(72.17) |
| DELTA | t | BH | 8.45(27.1) | 35(55.97) | 328.55(95.91) | 460.1(33.65) | 3.5(5.81) | 75(113.88) | 291.2(88.02) | 354.55(48.63) |
| DELTA | LIMMA | BONF | 0.7(2.15) | 3.75(9.72) | 117.8(72.85) | 295.7(55.94) | 0.9(1.29) | 16.6(36.4) | 121.8(82.95) | 209.55(71.66) |
| DELTA | LIMMA | BH | 22.35(60.21) | 55.35(74.75) | 336.25(94.71) | 461.95(33.54) | 29.55(39.71) | 97.4(119.84) | 298.05(86.34) | 358.4(46.96) |
| DELTA | Wilcox | BONF | 0(0) | 1.3(4) | 132.9(78.81) | 325.45(48.78) | 0(0) | 2.85(5) | 123.8(86.44) | 226.9(73.66) |
| DELTA | Wilcox | BH | 0(0) | 33.1(53.52) | 357(89.6) | 477(29.97) | 0(0) | 71.15(110.83) | 306.95(87.83) | 375.45(39.73) |
| DELTA | Nstat | BONF | 0(0) | 2.1(4.9) | 143.85(80.74) | 329.2(47.55) | 0(0) | 6(11.15) | 134.65(83.64) | 234.4(70.87) |
| DELTA | Nstat | BH | 0(0) | 37.85(60.65) | 355.5(90.74) | 475(29.73) | 0(0) | 80.8(115.99) | 311(81.16) | 374.8(39.69) |
| SVA | t | BONF | 1.25(4.45) | 13.8(28.86) | 396.7(189.31) | 642.2(127.84) | 0.35(0.93) | 22.75(48.88) | 292.15(132.89) | 418.7(87.49) |
| SVA | t | BH | 84.15(202) | 275.85(220.65) | 623.55(139.32) | 706.8(71.81) | 57.4(108.28) | 227.3(185.91) | 479.85(64.29) | 502.5(57.35) |
| SVA | LIMMA | BONF | 115.3(204.37) | 61.15(132.43) | 414.7(187.5) | 644.4(128.03) | 7.35(12.49) | 81.65(103.71) | 312.15(127.91) | 424.75(86.04) |
| SVA | LIMMA | BH | 235.25(292.35) | 334.3(235.51) | 625.95(140.33) | 706.95(72.34) | 137.8(160.13) | 269.7(170.25) | 483.4(61.45) | 502.65(58.09) |

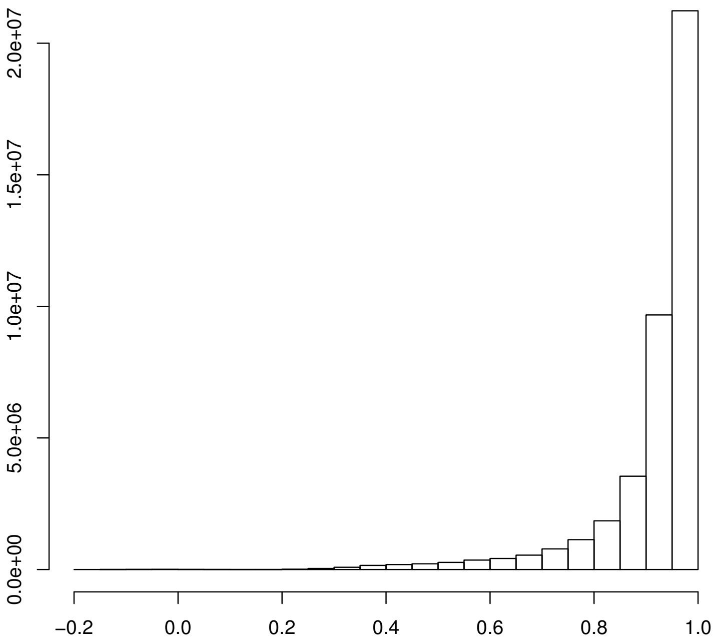


Figure S1: Histogram of pairwise Pearson correlation coefficients between genes computed from **HYPERDIP** without normalization. Number of genes: 9005. Number of arrays: 88.

# 4 Results of biological data analysis

Figure S1 provides a histogram of pairwise Pearson correlation coefficients computed from **HYPERDIP** without normalization. The sample mean of these correlation coefficients is 0.912.

Table S9 summarizes the results of biological data analyses. Most results in Table S9 agree with what we observe in the simulations. The results are conspicuous in that the numbers of total detected DEGs are very high for medium to large ($n = 30$ to $n = 79$) normalized datasets. Although it is hard to find out exactly how many of these genes are false discoveries, we think it is highly improper to have more than a thousand DEGs with 0.05 familywise error rate or four thousand DEGs (out of 9005 genes in total) with 0.05 false discovery rate. In other words, such a large number of positives most likely indicates the associated strategies failed to control FWER/FDR at the nominal level.

As observed in the simulation studies, gene selection strategies with normalization procedures detect more DEGs than those without normalization. When **BH** is applied in **HYPERDIP** vs. **TEL**, strategies with **GLOBAL** detect more positives than those with other normalization procedures. However, the strategies with **QUANTILE** and **RANK** detect more positives than those with **GLOBAL** when **BONF** is applied or the comparison is between **TALL** and **TEL**. This observation suggests that the technical noise may not be purely additive and is consistent with what we observe in **SIMU-BIO**. Among four normalization procedures, **DELTA** is the most conservative one in terms of the number of positives. Based on our simulation results, it is reasonable to believe that **DELTA** has relatively better control of type I errors.

By and large, the gene selection strategies based on $N$-statistics detect more positives than those based on the $t$-test and the Wilcoxon rank-sum test. More often than not, even the Wilcoxon rank-sum test detects more DEGs than the $t$-test. Just like in the **SIMU-BIO** study, this suggests that the expressions of biological data may not be normally distributed. Since tests based on $N$-statistic and Wilcoxon rank-sum statistic are both nonparametric, if the normality of data is in question, $N$-statistic can be used in place of

Table S9: The numbers of differentially expressed genes detected by different selection strategies. Total number of genes: 9005. Number of permutations for Nstat: 100000. The significance threshold: 0.05.

| | | | **HYPERDIP vs. TEL** | | | | **TALL vs. TEL** | | | |
|---|---|---|---|---|---|---|---|---|---|---|
| Norm | Test | MTP | n=5 | n=10 | n=40 | n=79 | n=5 | n=10 | n=30 | n=45 |
| NONE | t | BONF | 0 | 0 | 42 | 110 | 2 | 6 | 45 | 107 |
| NONE | t | BH | 0 | 0 | 125 | 314 | 6 | 7 | 111 | 230 |
| NONE | LIMMA | BONF | 17 | 52 | 99 | 147 | 32 | 26 | 94 | 164 |
| NONE | LIMMA | BH | 78 | 120 | 191 | 359 | 68 | 49 | 197 | 307 |
| NONE | Wilcox | BONF | 0 | 0 | 35 | 103 | 0 | 0 | 44 | 103 |
| NONE | Wilcox | BH | 0 | 0 | 101 | 308 | 0 | 6 | 98 | 226 |
| NONE | Nstat | BONF | 0 | 1 | 44 | 103 | 0 | 0 | 51 | 109 |
| NONE | Nstat | BH | 0 | 1 | 100 | 290 | 0 | 7 | 105 | 224 |
| GLOBAL | t | BONF | 1 | 10 | 358 | 901 | 7 | 76 | 641 | 924 |
| GLOBAL | t | BH | 2 | 422 | 2498 | 4032 | 36 | 438 | 2233 | 2633 |
| GLOBAL | LIMMA | BONF | 3 | 17 | 370 | 913 | 19 | 96 | 662 | 923 |
| GLOBAL | LIMMA | BH | 7 | 517 | 2496 | 4039 | 66 | 487 | 2233 | 2632 |
| GLOBAL | Wilcox | BONF | 0 | 0 | 394 | 959 | 0 | 0 | 702 | 978 |
| GLOBAL | Wilcox | BH | 0 | 408 | 2925 | 4083 | 0 | 452 | 2325 | 2718 |
| GLOBAL | Nstat | BONF | 0 | 3 | 429 | 1011 | 0 | 26 | 764 | 1031 |
| GLOBAL | Nstat | BH | 0 | 625 | 3117 | 4225 | 0 | 537 | 2707 | 2861 |
| QUANT | t | BONF | 1 | 15 | 383 | 1046 | 20 | 159 | 880 | 1229 |
| QUANT | t | BH | 1 | 205 | 1839 | 3381 | 209 | 858 | 2854 | 3344 |
| QUANT | LIMMA | BONF | 4 | 26 | 395 | 1048 | 60 | 215 | 910 | 1240 |
| QUANT | LIMMA | BH | 14 | 293 | 1848 | 3386 | 358 | 978 | 2865 | 3363 |
| QUANT | Wilcox | BONF | 0 | 0 | 418 | 1117 | 0 | 0 | 913 | 1266 |
| QUANT | Wilcox | BH | 0 | 210 | 2010 | 3627 | 0 | 820 | 2966 | 3474 |
| QUANT | Nstat | BONF | 0 | 3 | 449 | 1171 | 0 | 25 | 960 | 1318 |
| QUANT | Nstat | BH | 0 | 254 | 2047 | 3620 | 0 | 966 | 3073 | 3544 |
| RANK | t | BONF | 1 | 15 | 347 | 965 | 18 | 156 | 828 | 1172 |
| RANK | t | BH | 1 | 189 | 1711 | 3265 | 185 | 819 | 2799 | 3310 |
| RANK | LIMMA | BONF | 2 | 23 | 303 | 908 | 33 | 157 | 793 | 1130 |
| RANK | LIMMA | BH | 20 | 178 | 1615 | 3164 | 223 | 796 | 2697 | 3207 |
| RANK | Wilcox | BONF | 0 | 0 | 418 | 1117 | 0 | 0 | 913 | 1266 |
| RANK | Wilcox | BH | 0 | 210 | 2010 | 3627 | 0 | 820 | 2966 | 3474 |
| RANK | Nstat | BONF | 0 | 1 | 417 | 1135 | 0 | 17 | 932 | 1289 |
| RANK | Nstat | BH | 0 | 240 | 2007 | 3577 | 0 | 899 | 3062 | 3519 |
| DELTA | t | BONF | 0 | 4 | 147 | 406 | 3 | 35 | 360 | 548 |
| DELTA | t | BH | 0 | 84 | 972 | 1874 | 35 | 224 | 1364 | 2044 |
| DELTA | LIMMA | BONF | 0 | 6 | 156 | 412 | 18 | 47 | 368 | 551 |
| DELTA | LIMMA | BH | 2 | 140 | 975 | 1871 | 73 | 272 | 1389 | 2032 |
| DELTA | Wilcox | BONF | 0 | 2 | 170 | 425 | 0 | 27 | 370 | 598 |
| DELTA | Wilcox | BH | 0 | 64 | 1030 | 1842 | 0 | 230 | 1499 | 2204 |
| DELTA | Nstat | BONF | 0 | 3 | 198 | 488 | 0 | 32 | 416 | 663 |
| DELTA | Nstat | BH | 0 | 114 | 1136 | 1972 | 0 | 256 | 1718 | 2302 |
| SVA | t | BONF | 0 | 18 | 738 | 1619 | 7 | 94 | 1135 | 1356 |
| SVA | t | BH | 0 | 1582 | 3455 | 4256 | 937 | 1123 | 4601 | 4153 |
| SVA | LIMMA | BONF | 3 | 32 | 757 | 1619 | 26 | 143 | 1151 | 1375 |
| SVA | LIMMA | BH | 18 | 1843 | 3476 | 4255 | 1726 | 1235 | 4612 | 4139 |

$t$-test and Wilcox rank-sum test to achieve better statistical power.
